# Physical regulation of copper catalyst with a hydrophobic promoter for enhancing CO_2_ hydrogenation to methanol

**DOI:** 10.1016/j.xinn.2023.100445

**Published:** 2023-05-22

**Authors:** Hangjie Li, Wei Fang, Ling-Xiang Wang, Yifeng Liu, Lujie Liu, Tulai Sun, Ciqi Liao, Yihan Zhu, Liang Wang, Feng-Shou Xiao

**Affiliations:** 1 Key Lab of Biomass Chemical Engineering of Ministry of Education, College of Chemical and Biological Engineering, Zhejiang University, Hangzhou 310028, China; 2 Key Laboratory of Applied Chemistry of Zhejiang Province, Department of Chemistry, Zhejiang University, Hangzhou 310028, China; 3 Center for Electron Microscopy, State Key Laboratory Breeding Base of Green Chemistry Synthesis Technology, College of Chemical Engineering, Zhejiang University of Technology, Hangzhou 310014, China

## Abstract

The hydrogenation of CO_2_ to methanol, which is restricted by water products, requires a selective removal of water from the reaction system. Here, we show that physically combining hydrophobic polydivinylbenzene with a copper catalyst supported by silica can increase methanol production and CO_2_ conversion. Mechanistic investigation reveals that the hydrophobic promoter could hinder the oxidation of copper surface by water, maintaining a small fraction of metallic copper species on the copper surface with abundant Cu^δ+^, resulting in high activity for the hydrogenation. Such a physically mixed catalyst survives the continuous test for 100 h owing to the thermal stability of the polydivinylbenzene promoter.

## Public summary

• Hydrophobic polydivinylbenzene (PDVB) is physically mixed with a Cu/SiO_2_ catalyst.
• The PDVB improves the performance of the Cu/SiO_2_ catalyst in CO_2_ hydrogenation.
• The PDVB stabilizes the Cu^0^ sites against oxidation by water during the catalysis.
• The Cu^0^ and Cu^δ+^ sites synergistically catalyze the CO_2_ hydrogenation to methanol.

## Graphical abstract

**Figure:**
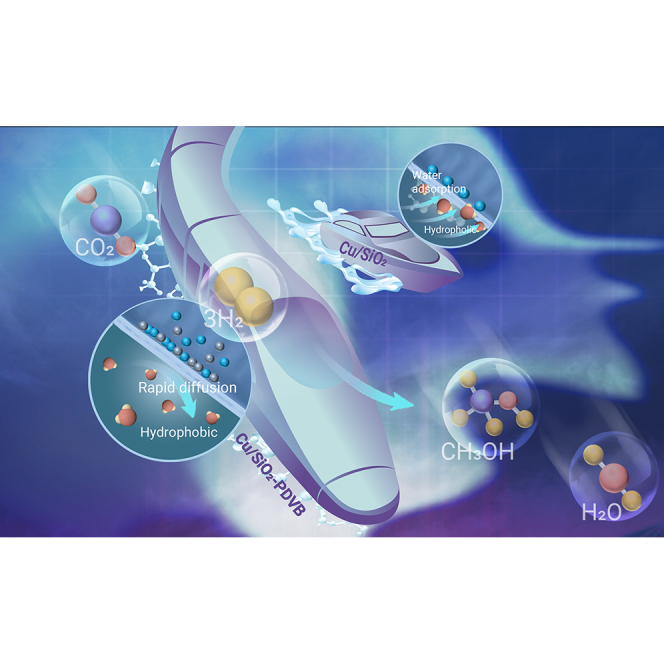

## Introduction

The hydrophobic water conduction channels have displayed a crucial role in enzyme catalysis, which rapidly ships the water products from the active sites to accelerate the reactions.^1^ Following enzyme catalysis, this function has been introduced to the heterogeneous catalysts by functionalizing the catalyst surface with self-assembled molecular monolayers or organosilanes.^2^^,^^3^^,^^4^^,^^5^^,^^6^^,^^7^^,^^8^^,^^9^ In many cases, these molecules would block the catalyst surface to partially lose the active sites and suffer from thermal instability under the stream at high temperatures. In contrast with these chemical modification methods that might change the catalyst surface, the zeolite membrane reactors^3^ with water conduction channels are ideal for efficiently shipping water molecules, where the catalyst surface is unscathed. However, there are still great challenges in synthesizing extensive zeolite membranes in industrial processes.

Recently, we developed an efficient strategy for rapidly removing the water product from the surface of metal carbide catalysts to accelerate the Fischer-Tropsch synthesis to olefins,^5^ which is achieved by physically mixing the catalysts with a nonporous hydrophobic polydivinylbenzene (PDVB). In this case, the catalyst was unchanged relative to the catalyst with chemical modification, which can be denoted as a physical regulation strategy. This success motivated the exploration of whether this strategy could be fabricated to promote the challenging reactions severely restricted by water both thermodynamically and kinetically, such as hydrogenation of CO_2_ to methanol, which is an important reaction for the production of valuable platform chemicals from CO_2_.^10^^,^^11^^,^^12^^,^^13^^,^^14^^,^^15^^,^^16^^,^^17^ Generally, the water product on the catalyst could oxidize the metal surface to partially lose the activity.^18^^,^^19^ Despite the fact that the approaches utilizing a zeolite membrane reactor have been successful in this process,^3^ a reliable method that is simple to implement and completely unaffected to the present catalysts is still urgently needed. In this work, we demonstrated that the hydrophobic promoter mixed with silica-supported copper catalyst would influence the oxidation state of the Cu catalyst, thus enhancing the performances in the hydrogenation of CO_2_ to methanol. Such a change in the chemical state led by physical regulation might guide the catalyst design in heterogeneous catalysis.

## Results and discussion

### Structural characterization and catalytic performance

**Figure 1:**
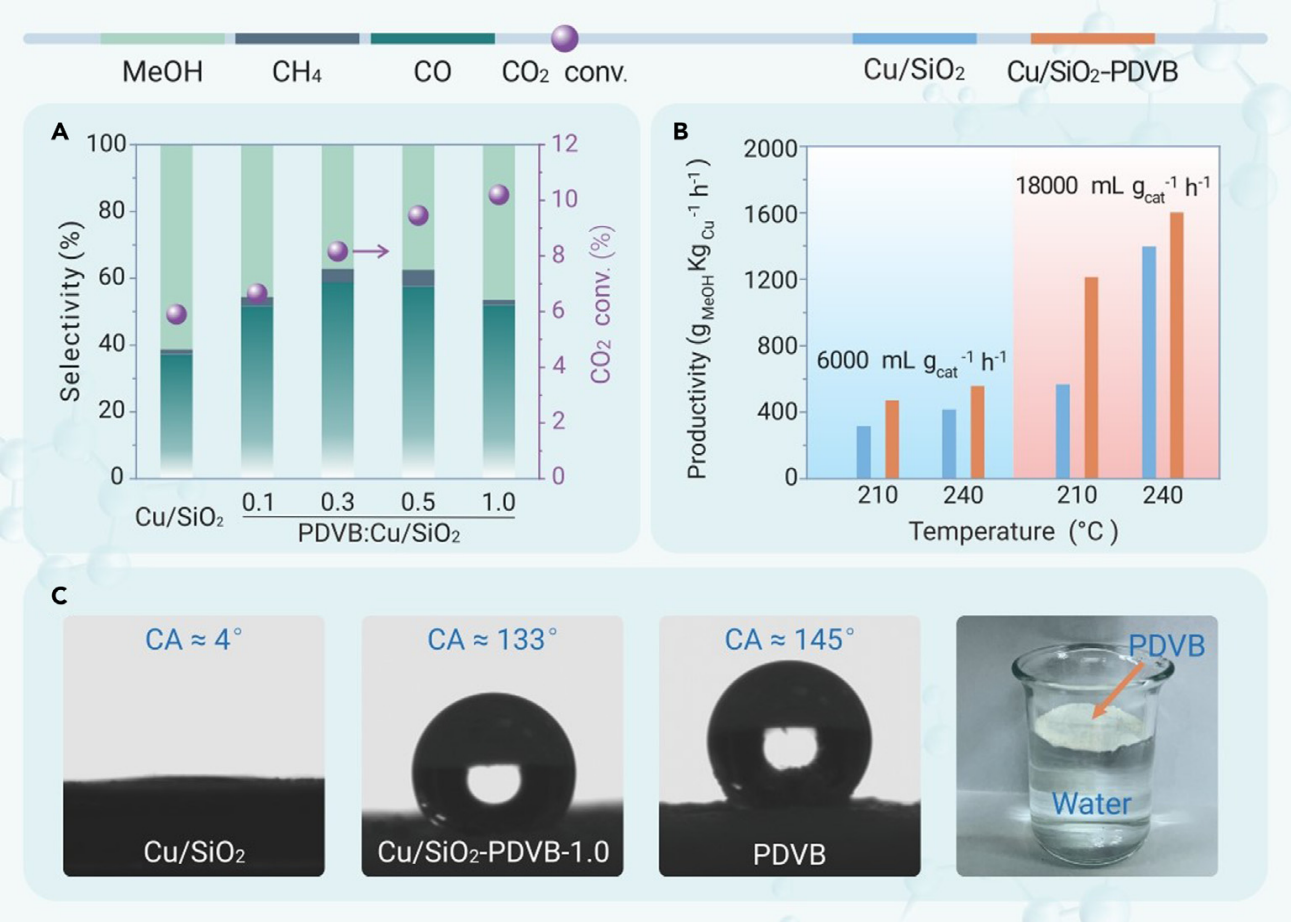
Catalytic data in CO2 hydrogenation (A) Data showing the catalytic performance of the Cu/SiO_2_-PDVB with different weight ratios of PDVB to Cu/SiO_2_ in CO_2_ hydrogenation. Reaction conditions: 3 MPa, 240°C, SV of 6,000 mL g_cat_^−1^ h^−1^, H_2_/CO_2_/Ar ratio at 72/24/4 vol %. Cu/SiO_2_-PDVB represents the catalyst in powder mixing manner (the Cu/SiO_2_ powder was mixed with an equivalent weight of PDVB powder and then squeezed and crushed into granules with 20–40 mesh size for tests). The Cu/SiO_2_ granules at 20–40 mesh without PDVB were used as a reference. (B) Data showing the methanol productivity of the Cu/SiO_2_ and Cu/SiO_2_-PDVB catalysts in CO_2_ hydrogenation. Reaction conditions: 3 MPa, 210°C, SV of 6,000 and 18,000 mL g_cat_^−1^ h^−1^, or 3 MPa, 240°C, SV of 6,000 and 18,000 mL g_cat_^−1^ h^−1^, H_2_/CO_2_/Ar ratio at 72/24/4 vol %. (C) Water droplet contact angles of the Cu/SiO_2_, Cu/SiO_2_-PDVB, PDVB, and the photograph showing PDVB floating on the water.

In the proof-of-concept experiment, we physically mixed the hydrophobic and nonporous PDVB (surface area <5 m^2^ g^−1^, water droplet contact angle at ∼145°, Figure 1) with the silica-supported copper catalyst (Cu/SiO_2_, Cu loading amount at 16.0 wt %), a well-known catalyst for CO_2_ hydrogenation to methanol. Data characterizing the performances of various catalysts under the given reaction conditions (3 MPa, 240°C, 6,000 mL g_cat_^−1^ h^−1^) are shown in Figure 1A. In these tests, the CO_2_ conversion and methanol selectivity were below the equilibrium,^20^ which can be safely treated to represent to the reaction rate. The blank run without catalysts failed to transform CO_2_. The Cu/SiO_2_ (surface area as ∼222 m^2^ g^−1^, water droplet contact angle at 4°, Figures 1C, S1, and S2) catalyzed the reaction with CO_2_ conversion and methanol selectivity at 5.9% and 61.3%, respectively, which are similar to those of the silica-supported copper catalysts tested previously.^21^ In this case, the methanol productivity was ∼420 g_MeOH_ kg_Cu_^−1^ h^−1^. After mixing the Cu/SiO_2_ catalyst with hydrophobic PDVB (Cu/SiO_2_-PDVB) in a powder mixing manner (the Cu/SiO_2_ and PDVB powder were mixed together and then granulated for the catalytic test), the CO_2_ conversion was raised to 6.6%–10.2%. Although the methanol selectivity was decreased because of the simultaneously accelerated reverse water-gas shift by the hydrophobic promoter, the enhanced methanol productivity was achieved by optimizing the PDVB amount (weight ratio of PDVB to Cu/SiO_2_ at 1.0, water droplet contact angle at 133°), giving ∼558 g_MeOH_ kg_Cu_^−1^ h^−1^ (CO_2_ conversion at 10.2%, methanol selectivity at 46.5%), which steadily outperforms that without PDVB. These results confirm the promotion effect of PDVB on the reaction (Figures 1B and S3, Table S1).

The CO_2_ conversion, methanol selectivity, and gas feeding rate determine the methanol productivity. In the tests at lower temperatures and higher gas feeding rates, the methanol selectivity could be further improved (Table S2 and Figure S4).^22^ For example, at 210°C with a gas feeding rate of 18,000 mL g_cat_^−1^ h^−1^, the methanol selectivity was 86.0% with CO_2_ conversion at 4.0% over the Cu/SiO_2_-PDVB catalyst, resulting in the methanol productivity at ∼1,213 g_MeOH_ kg_Cu_^−1^ h^−1^ (Table S2). Further increasing the reaction temperatures to 230°C and 240°C led to the methanol productivities at 1,454 and 1,602 g_MeOH_ kg_Cu_^−1^ h^−1^, respectively. These data are higher than that of the Cu/SiO_2_ catalysts under the equivalent conditions. By studying the performances of Cu/SiO_2_ and Cu/SiO_2_-PDVB catalysts under a scope of temperatures and gas feeding rates, the PDVB could always improve the performance of Cu/SiO_2_ catalyst under multiple conditions (Table S2 and Figure S4), and such enhancement effect was more obvious at lower temperatures (Figures 1B, S5, and S6).

**Figure 2:**
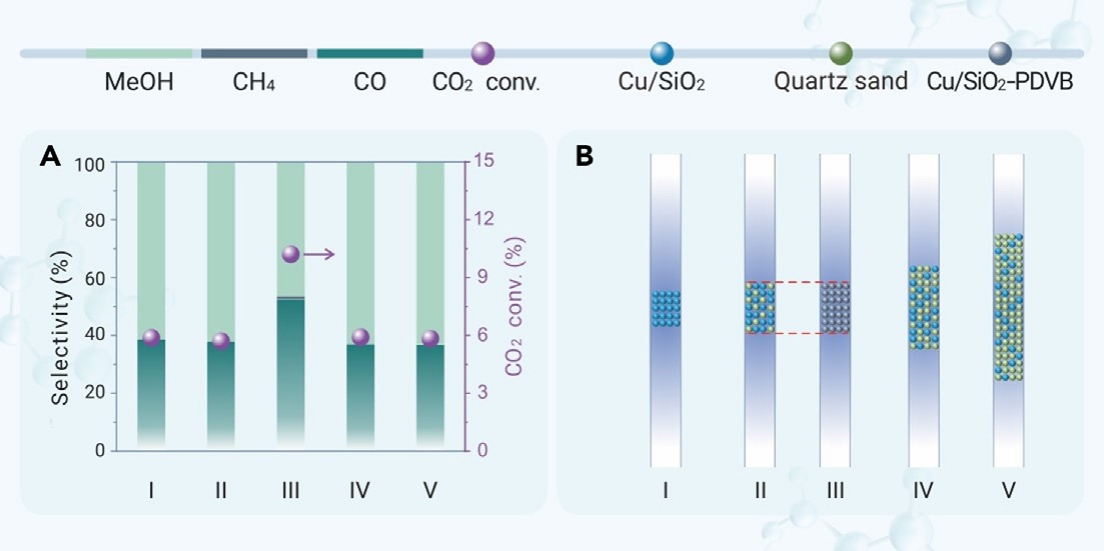
Influence of mixing manners to the catalysis Data showing (A) the catalytic performance and (B) the schemes for mixing manner of the Cu/SiO_2_, Cu/SiO_2_-quartz sand, and Cu/SiO_2_-PDVB catalysts in CO_2_ hydrogenation. Reaction conditions: 3 MPa, 240°C, SV of 6,000 mL g_cat_^−1^ h^−1^, H_2_/CO_2_/Ar ratio at 72/24/4 vol %. The SV (mL g_cat_^−1^ h^−1^) was calculated according to the weight of Cu/SiO_2_ catalyst amount in the reactor, and the PDVB promoter and inert quartz sand were not considered. Cu/SiO_2_-PDVB (Entry III) represents the catalyst in powder mixing manner (the Cu/SiO_2_ powder was mixed with an equivalent weight of PDVB powder and then squeezed and crushed into granules with 20–40 mesh size for tests). The manners of the catalyst beds were shown in the schemes. Entry I, 0.2 g of Cu/SiO_2_ granule; Entry II, 0.2 g of Cu/SiO_2_ granule mixed with 0.1 g of quartz sand granule; Entry IV, 0.2 g of Cu/SiO_2_ granule mixed with 3.0 g of quartz sand granule; Entry V, 0.2 g of Cu/SiO_2_ granule mixed with 6.0 g of quartz sand granule. All granules used in the reactions are in 20–40 mesh size.

The PDVB diluted the Cu/SiO_2_ component of the Cu/SiO_2_-PDVB catalyst. One would expect that this changed the residence time in the total catalyst bed compared with the PDVB-free catalyst, which might influence the catalysis. In order to exclude this issue, we diluted the Cu/SiO_2_ catalyst with inert quartz sands (Cu/SiO_2_-quartz sand) to obtain the same volume of catalyst bed as Cu/SiO_2_-PDVB catalyst. The Cu/SiO_2_ and Cu/SiO_2_-quartz sand exhibited almost the same CO_2_ conversion, methanol selectivity, and methanol productivity (I and II in Figure 2). Even diluting the Cu/SiO_2_ catalyst with more quartz sands exhibiting a larger total volume than the Cu/SiO_2_-PDVB catalyst, the performances were still similar to those of the bare Cu/SiO_2_ without any diluter (IV and V in Figure 2), confirming the insensitivity of inert diluter to the performances (Figure S5). This result is due to that the efficient residence time on the Cu/SiO_2_ component was negligibly influenced by the amount of diluter, which is in good agreement with the previous phenomena.^12^ In addition, we further compared the performances of Cu/SiO_2_-quartz sand and Cu/SiO_2_-PDVB catalysts with the same catalyst packing volume under multiple reaction conditions (Figure S5). As a result, the Cu/SiO_2_-PDVB always exhibited higher methanol productivity relative to Cu/SiO_2_-quartz sand, which suggests that the PDVB indeed enhanced the performances by the hydrophobicity (Figure S5), while this effect was undetected on the hydrophilic quartz diluter.

Additionally, we hydrophobized the Cu/SiO_2_ catalyst using an organosilane of dimethyl diethyloxysilane (Cu/SiO_2_-Me), a conventional chemical modification route for achieving a hydrophobic surface.^6^ By varying the contents of organosilane at 10 wt % and 30 wt % on the Cu/SiO_2_-Me, the resulting catalysts gave CO_2_ conversions at 6.2% and 5.3% with methanol selectivities at 53.2% and 59.8%, respectively (Figure S7, Table S3). Water droplet contact angles are ∼41° and ∼151° for these samples (Figure S8). Particularly, the Cu/SiO_2_-Me with an organosilane content of 30% was even more hydrophobic than the Cu/SiO_2_-PDVB catalyst but exhibited a relatively lower CO_2_ conversion. This phenomenon should be due to that the catalyst surface was blocked by the organosilane layer (Figures S9–S11).

**Figure 3:**
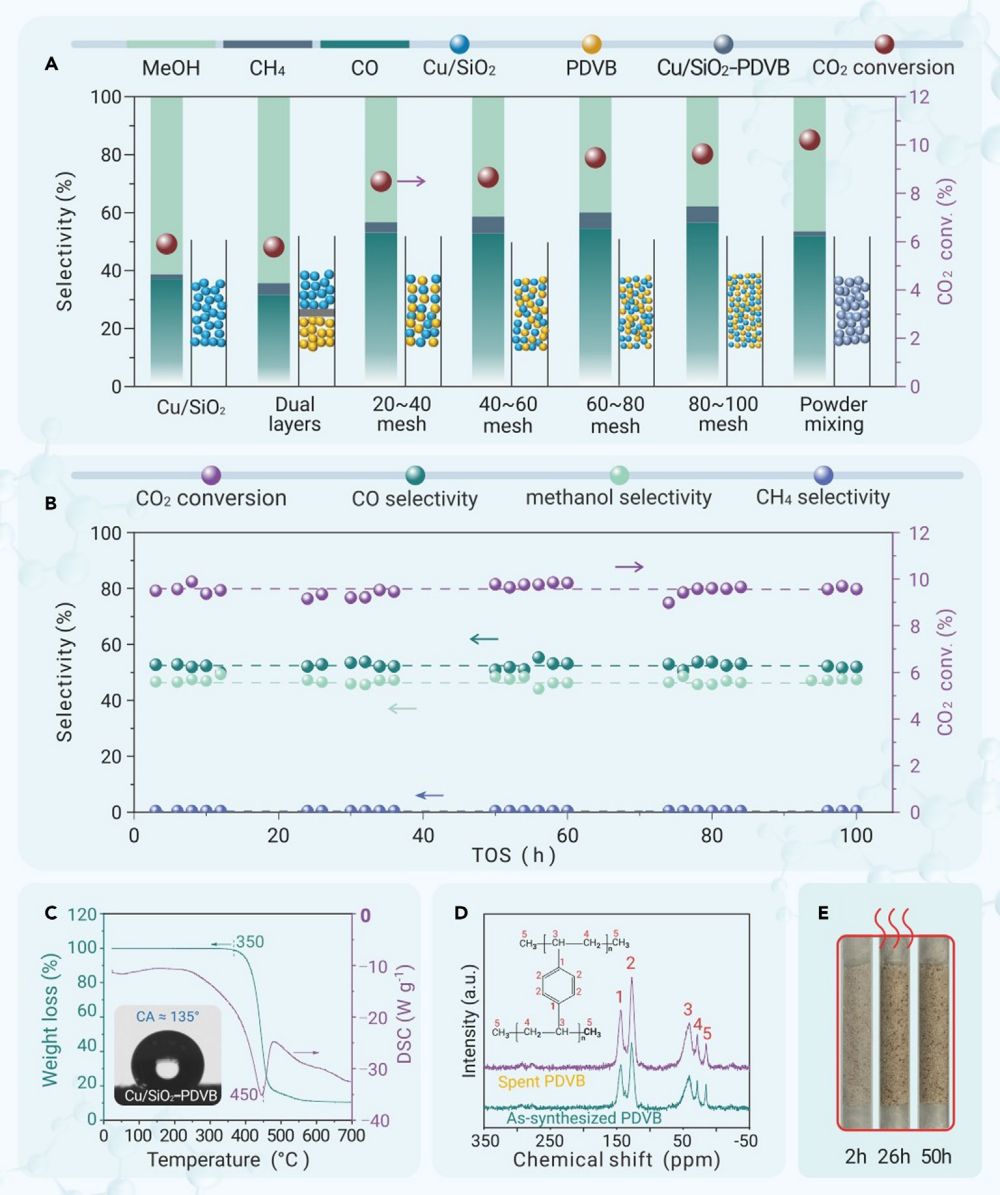
The evaluation data of CO2 hydrogenation and stability tests Data showing (A) Catalytic performance of the Cu/SiO_2_-PDVB with different mixing manners between the Cu/SiO_2_ and PDVB in CO_2_ hydrogenation. Reaction conditions: 3 MPa, 240°C, SV of 6,000 mL g_cat_^−1^ h^−1^, H_2_/CO_2_/Ar at 72/24/4 vol %. (B) Durability test of the Cu/SiO_2_-PDVB catalyst in CO_2_ hydrogenation. Reaction conditions: 3 MPa, 240°C, 6,000 mL g_cat_^−1^ h^−1^, H_2_/CO_2_/Ar ratio at 72/24/4. (C) TG-DSC profiles of the PDVB. Inset, water droplet contact angle of spent Cu/SiO_2_-PDVB. (D) ^13^C-NMR spectra of the PDVB component of as-synthesized and spent Cu/SiO_2_-PDVB. (E) Photographs showing the PDVB granules in a quartz tube with thermal treatment at 240°C for different periods.

To reveal the function of PDVB during the catalysis, we reasonably adjusted its mixing manners with Cu/SiO_2_ catalyst. The data characterizing the performances are shown in Figure 3A. The catalyst in a dual-bed manner with PDVB localized under Cu/SiO_2_ in separated beds resulted in a CO_2_ conversion of 5.8% and methanol selectivity of 64.4%, which is similar to those of the bare Cu/SiO_2_ catalyst without PDVB. For the catalysts with granule mixing manners (the Cu/SiO_2_ and PDVB were made into granules separately and then mixed in the bed for catalysis), the CO_2_ conversions were enhanced, giving 8.5%, 8.7%, 9.5%, and 9.6% for the catalysts with granule sizes at 20–40, 40–60, 60–80, and 80–100 meshes, respectively. In these cases, the methanol selectivities were 37.9%–43.3%. In the powder mixing manner, the CO_2_ conversion reached 10.2% with methanol selectivity at 46.5%. Considering the performances of a sole Cu/SiO_2_ catalyst without PDVB were not sensitive to the granule sizes (Figure S12), these results suggest that the proximity would benefit the promotion effect of PDVB, in good agreement with our previous results in Fischer-Tropsch synthesis to olefins.^5^ For the Cu/SiO_2_ granules mixed with PDVB granules (20–40 mesh), the CO_2_ conversion was obviously enhanced relative to the Cu/SiO_2_ granules mixed with quartz sands (Figures 2A and S5), which should be reasonably due to the different wettability of the PDVB and quartz sands.

The durability of the Cu/SiO_2_-PDVB catalyst was evaluated in a continuous reaction test. The results are shown in Figure 3B. In 100 h, the CO_2_ conversions and methanol selectivities were constant at ∼9.7% and ∼47.0%, respectively. These data evidence the good durability of Cu/SiO_2_-PDVB for the CO_2_ hydrogenation to methanol. One may anticipate that the PDVB would melt or break down to alter the Cu nanoparticles' intrinsic activity. By confirming the excellent stability of PDVB, we ruled out this hypothesis. Figure 3C shows the thermogravimetric-differential scanning calorimetry (TG-DSC) profiles of PDVB that gave weight loss starting at 380°C, suggesting the stable PDVB at the reaction temperature of 240°C for CO_2_ hydrogenation. The spent Cu/SiO_2_-PDVB catalyst showed the water droplet contact angle at 135°C, which is similar to that of the fresh catalyst (inset in Figure 3C). The stability of the polymer network of PDVB was further explored by the ^13^C NMR characterizing the PDVB component in Cu/SiO_2_-PDVB catalysts before and after the tests in CO_2_ hydrogenation. As shown in Figure 3D, the PDVB component in both catalysts showed similar signals assigning to the carbon species on the aromatic ring (143 and 126 ppm) and aliphatic chain (40.3, 28.6, and 14.5 ppm).^5^ These data demonstrate the stable PDVB under the reaction conditions, which is further supported by the FTIR characterization (Figure S13). To further evaluate the stability at the reaction temperature, we heated PDVB at 240°C, and the effluent was analyzed by mass spectroscopy (Figure S14). The possible species from PDVB decomposition were completely undetectable. Figure 3E shows the photographs of the PDVB granules during heating treatment at 240°C and 300°C for different periods (Figures S15 and S16), giving the maintained granule shape to exclude the possibility of its melting and flowing in the reactor. This hypothesis was also supported by the SEM characterizations in Figure S17, which showed similar morphology of the PDVB component in the spent Cu/SiO_2_-PDVB catalyst to that of the fresh catalyst. To further confirm the PDVB-promoted process, we removed the PDVB component from the spent Cu/SiO_2_-PDVB catalyst and evaluated the resulting Cu/SiO_2_ component in CO_2_ hydrogenation, exhibiting similar performances to that of the as-prepared Cu/SiO_2_ catalyst (Figure S18).

We explored the performance of other materials with different wettability in promoting the catalysis over Cu/SiO_2_, including the hydrophobic materials of polyacrylonitrile (PAN, water droplet contact angle at 63°, Figure S19), polyamide (PA, water droplet contact angle at 82°), polytetrafluoroethylene (PTFE, water droplet contact angle at 117°), and hydrophilic materials (Figure S20, water droplet contact angles <5°) of amorphous silica, anatase, silanol-rich siliceous MFI zeolite (S-1, Figure S21). All these materials influenced the catalysis, giving improved CO_2_ conversions of 7.3%, 9.2%, and 10.1% with some loss of methanol selectivity over the Cu/SiO_2_ catalysts with PAN, PA, and PTFE promoters, respectively (Figure S22). With these hydrophilic promoters, the CO_2_ conversions were only 2.0%–5.6% (Figure S23). These data confirm the crucial role of promoter wettability for catalysis, where the hydrophobic promoter could realize enhanced performances.

### Active sites and reaction mechanism

**Figure 4:**
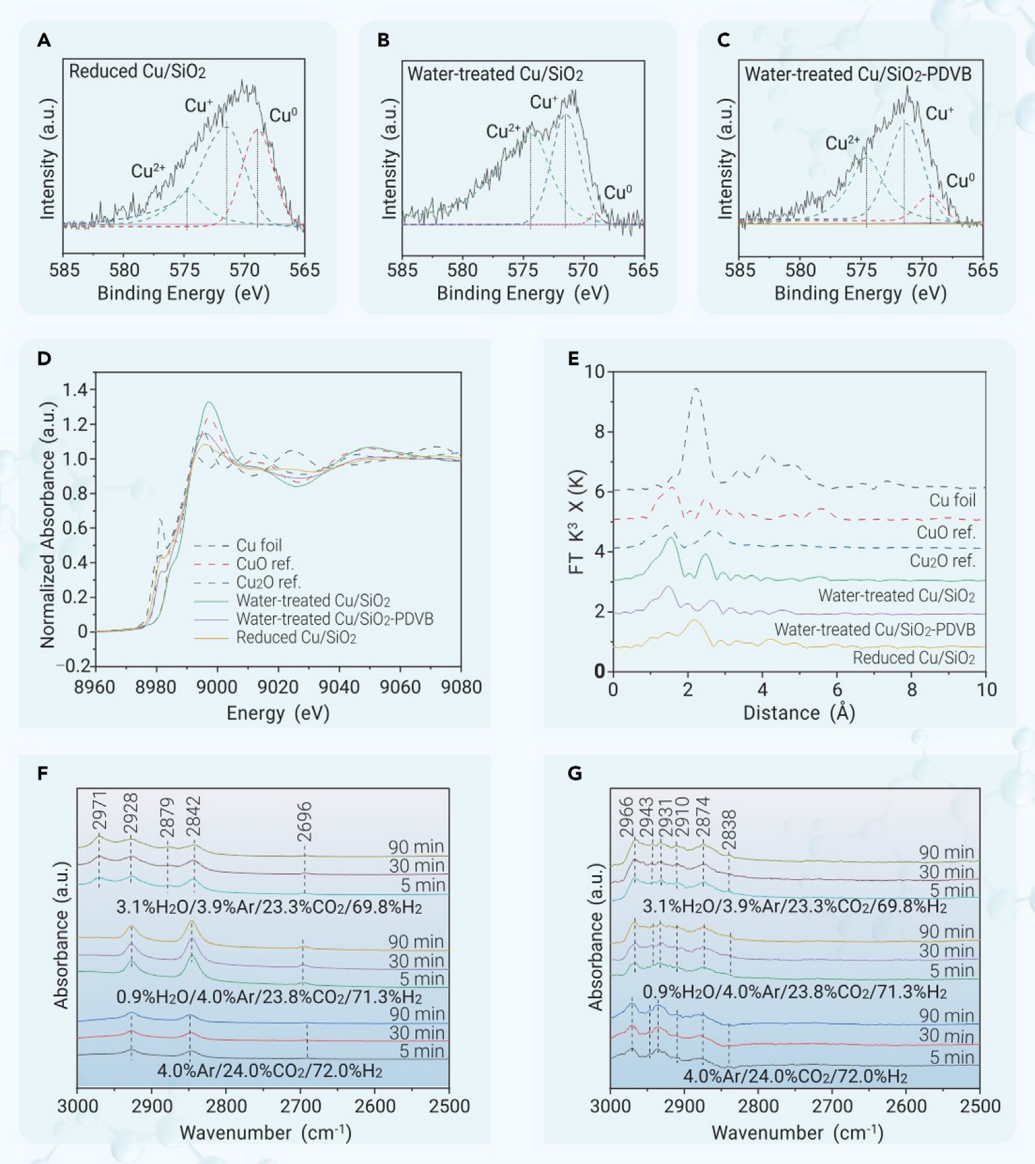
The evaluation data of CO2 hydrogenation and stability tests Cu LMM Auger XPS spectra of (A) reduced Cu/SiO_2_ with hydrogen, (B) water-treated Cu/SiO_2_, and (C) water-treated Cu/SiO_2_-PDVB. Cu K-edge (D) XANES and (E) EXAFS spectra in the R space of Cu/SiO_2_ and Cu/SiO_2_-PDVB catalysts, without correcting for scattering phase shift. *In situ* DRIFT spectra of (F) Cu/SiO_2_ and (G) Cu/SiO_2_-PDVB in contact with H_2_/CO_2_/Ar (72/24/4, vol %) at 240°C.

The Cu/SiO_2_-PDVB always gave higher CO_2_ conversion and methanol productivity than the Cu/SiO_2_ at different reaction temperatures (Figures S24 and S25). The apparent activation energies (E_a_) of the Cu/SiO_2_ and Cu/SiO_2_-PDVB catalyzed CO_2_ hydrogenation were 69.7 and 35.1 kJ mol^−1^, respectively (Figure S26). Obviously lower apparent E_a_ supported an easier reaction on the Cu/SiO_2_-PDVB catalyst than that on the Cu/SiO_2_. It has been previously identified that high water partial pressure (eg 10^5^ Pa) would oxidize the copper surface, which explains the suppressed activity by water in the previous reaction systems.^18^^,^^19^^,^^23^ In the Cu/SiO_2_ catalyzed CO_2_ hydrogenation, the partial pressure of water in the reactor was ∼4.4 ∗ 10^4^ Pa, which increased to ∼7.8 ∗ 10^4^ Pa over the Cu/SiO_2_-PDVB catalyst because of higher CO_2_ conversions (Table S4). The oxidation of copper by water was characterized by CO adsorption FTIR (Figure S27). The spectrum of as-reduced Cu/SiO_2_ showed the CO adsorption signals at 2,045 and 2,125 cm^−1^, which are assigned to the metallic Cu^0^ and cationic Cu^δ+^ species, respectively,^24^^,^^25^ in good agreement with the general feature of silica-supported Cu catalysts.^20^^,^^21^ Both Cu^δ+^ and Cu^0^ sites are required for CO_2_ hydrogenation, because the former benefits the CO_2_ adsorption, CO_2_ activation (Figure S28), and stabilization of reaction intermediates, while the latter could accelerate the hydrogenation of reaction intermediates.^20^^,^^21^ After a steam treatment, the Cu^0^ signal disappeared on Cu/SiO_2_ but still existed on Cu/SiO_2_-PDVB. This feature was further characterized by the Cu LMM Auger XPS spectra characterizing the as-reduced and water-treated Cu samples. The as-reduced Cu/SiO_2_ shows the signals assigned to Cu^0^, Cu^+^, and Cu^2+^ (Figure 4A), in agreement with the general phenomenon.^26^ After a steam treatment at 240°C for 24 h, the Cu was obviously oxidized with negligible Cu^0^ signal and obviously enhanced Cu^+^/Cu^2+^ signals (Figure 4B). Interestingly, the Cu/SiO_2_-PDVB showed superior oxidation resistance, as confirmed by the XPS spectrum of water-treated Cu/SiO_2_-PDVB with well-maintained Cu^0^ signal (Figure 4C).

The X-ray absorption spectra characterized the average structure information of overall Cu species. Figures 4D and 4E showed the spectra of X-ray absorption near edge structure (XANES) and extended X-ray absorption fine structure (EXAFS) of as-reduced Cu/SiO_2_, water-treated (240°C, 24 h) Cu/SiO_2_, and Cu/SiO_2_-PDVB. The as-reduced Cu/SiO_2_ exhibited Cu *K*-edge XANES spectrum with an adsorption edge between those of the Cu foil and Cu_2_O. This result suggests the dominant metallic copper, which is supported by the obvious metallic Cu-Cu distance in the EXAFS spectrum. After the steaming treatment, the Cu/SiO_2_ showed the XANES spectrum close to that of the reference CuO, confirming the oxidation of metallic Cu into CuO. The reduced Cu/SiO_2_ catalyst gave the shell distances at ∼1.78 and 2.50 Å, which are derived from the Cu-O and Cu-Cu, as evidenced by the referenced samples.^27^ After the pretreatment with water, the shell distances of Cu/SiO_2_ turn into ∼1.87 and 2.81 Å, which were identified for Cu-O and Cu-Cu in CuO, suggesting the oxidation of Cu species.^28^ However, the water pretreated Cu/SiO_2_-PDVB catalyst showed shell distances at ∼1.80 and 2.98 Å, which were corresponding to the Cu−O and Cu−Cu in Cu_2_O,^27^ suggesting a lower valence state of Cu species, in good agreement with the results of XANES spectrum. These data confirm the partially hindered Cu oxidation with water by physically mixing Cu/SiO_2_ with PDVB, resulting in the catalyst with Cu^0^ and Cu^δ+^, which are both required for the efficient CO_2_-to-methanol conversion.^21^ In contrast, the general Cu/SiO_2_ catalyst would be easily oxidized by water to partially lose the activity, which is in good agreement with the previous theoretical studies on water-deactivated copper in hydrogenations.^18^^,^^29^

Although the reaction pathways of the CO_2_-to-methanol process are still controversial, the formate route has been generally accepted on Cu-based catalysts.^30^^,^^31^^,^^32^ Because of the stability of the formate intermediate, it usually blocks the catalyst surface to hinder the reactions. Further insights for distinguishing the Cu/SiO_2_ and Cu/SiO_2_-PDVB catalyzed processes were performed using the *in situ* FTIR (Figures 4F and 4G). After inducing CO_2_ and hydrogen to the Cu/SiO_2_ catalyst, the signals at 2,928, 2842, and 2,696 cm^−1^ appeared, which are assigned to the methoxyl, formate, and carbonate species, respectively (Figure 4F).^33^^,^^34^ When a slight amount of water was introduced (0.9% and 3.1% in the feed with partial pressures of ∼0.9 x 10^3^ and ∼3.1 x 10^3^ Pa, respectively), the formate (2,971, 2,879, and 2,842 cm^−1^) signals were obviously enhanced,^35^ confirming the accelerated formation of formate species, but its further transformation was hindered. This result is constant with the general knowledge of water-promoted CO_2_ activation and primary hydrogenation.^35^^,^^36^ The formate accumulation on the catalyst surface would block the active sites for further reaction.^37^

Interestingly, introducing CO_2_ and hydrogen to the Cu/SiO_2_-PDVB catalyst failed to give formate species in the *in situ* FTIR study, but it exhibited the signals at 2,966, 2,931, 2,910, 2,874, 2,838, 1,362, 1,056, 1,032, and 1,007 cm^−1^, which are assigned to the methoxyl and methanol species (Figures 4G and S29).^35^ The signals at 1,410 and 1,387 cm^−1^ are assigned to carbonate species.^38^ These results might be due to the oxidation-resistant metallic Cu on the Cu/SiO_2_-PDVB catalyst, which benefits the rapid hydrogenation of formate, an important step in CO_2_-to-methanol conversion.^39^^,^^40^ Even after introducing water to the Cu/SiO_2_-PDVB catalyst with CO_2_ and hydrogen, the formate signals were still undetectable, suggesting water resistance.

A study on the side reaction was performed in the methanol decomposition as a model, which usually reduces the methanol selectivity to form CO. Figure S29 showed the performances of the Cu/SiO_2_ and Cu/SiO_2_-PDVB in the direct decomposition of methanol, giving the methanol conversions at 36.0% and 13.1%, respectively (Figure S30). In the methanol decomposition containing water, which simulates the reaction atmosphere in the CO_2_ hydrogenation, the Cu/SiO_2_ exhibited methanol conversion at 43.9% in the initial test, and then it continuously decreased to 14.1% after 6 h (Figure S30B). Compared with the as-synthesized Cu/SiO_2_, the FTIR spectrum of the spent catalyst showed additional signals at 3,676 and 3,656 cm^−1^ (Figure S31), assigning to the Cu-related hydroxyl species.^41^^,^^42^ These hydroxyl species would oxidize the Cu^0^ species on copper nanoparticles to deactivate the catalyst, which results in lower activity but higher methanol selectivity in CO_2_ hydrogenation. In the equivalent test, the Cu/SiO_2_-PDVB exhibited constant methanol conversions (28.8%–31.2%) during the test without deactivation, different from that of the Cu/SiO_2_ catalyst (Figure S30B). FTIR spectrum of the spent Cu/SiO_2_-PDVB showed undetectable signals of Cu-related hydroxyl species (Figure S31), confirming the resistance against oxidation by water after mixing with PDVB. The stable metallic Cu species would improve the activity for both CO_2_ hydrogenation^43^^,^^44^ and methanol decomposition (Figure S32), which explained the enhanced CO_2_ conversion and reduced apparent *E*_*a*_ (Figure S33) but partially lost methanol selectivity of PDVB-promoted catalyst in the CO_2_ hydrogenation.

Based on these results, we proposed a model showing the function of hydrophobic PDVB to promote CO_2_ hydrogenation. The PDVB showed irregular morphology with sizes at several to a few hundred micrometers. In contrast, the Cu/SiO_2_ was much smaller, having sizes at 100–300 nm. For the physical mixture of PDVB and Cu/SiO_2_, the small Cu/SiO_2_ was dispersed on the bulky PDVB matrix (Figure S34). This is because the PDVB negligibly hinders the adsorption of CO_2_ and hydrogen, which could access the Cu nanoparticles easily and make the hydrogenation reactions occur. Once the water was formed, most of them would escape from the catalyst surface because of the hydrophobic environment constructed by the PDVB individuals, as observed in our previous study.^5^ In addition, we also designed experiments to study the water diffusion in the PDVB-containing fixed bed and to simulate the water re-adsorption on the hydrophilic catalyst. As shown in Figure S35, we fixed the powder mixture of PDVB and copper sulfate anhydrate (CuSO_4_, as a color indicator to water) in the quartz tube and then purged by 30 vol % H_2_O/N_2_ with a rate at 25 mL min^−1^. For comparison, a mixture of CuSO_4_ and quartz powder was tested under equivalent conditions. The color change was recorded with time to represent the diffusion behavior of water. In the two cases, the test with PDVB showed a faster but lighter color change, while the test with quartz powder showed a slower color change, but the color was relatively darker. These data also support that the hydrophobic PDVB would accelerate the diffusion of water through the fixed bed, while the hydrophilic quartz leads to the accumulation of water. Further study was performed by localizing the two mixtures in a wet atmosphere, and the mixture containing PDVB still exhibited almost unchanged white color after 36 h, while the mixture containing quartz showed obvious blue color after only 12 h, indicating that the PDVB hindered water adsorption to the CuSO_4_ by a physically mixing (Figure S36). All these results reveal that physically mixing PDVB with Cu catalyst could accelerate the water desorption/diffusion from the catalyst bed (Figure S37) and could avoid water accumulation on the hydrophilic catalyst surface (Figure S38).^45^ The addition of PDVB would stabilize the small fraction of metallic Cu^0^ sites on the catalyst against oxidation by water, which benefits maintaining the active Cu^0^-Cu^δ+^ surface for the hydrogenation of CO_2_ to methanol (Figures S38–S41).

## Materials and methods

See supplemental information for details.

## Conclusion

In sum, we have demonstrated a hydrophobic promoter to accelerate the CO_2_ hydrogenation to methanol through a physical regulation strategy. By physically mixing the hydrophobic promoter with the supported Cu nanoparticle catalyst, CO_2_ conversion and methanol productivity could be efficiently improved. Considering the facile operation in the physical mixing strategy, it might be useful to optimize more catalysts in CO_2_ hydrogenation, such as the Cu-ZnO-Al_2_O_3_ catalysts involved in this research (Tables S5–S8).

## Lead contact website

http://www.chem.zju.edu.cn/xiaofs/main.htm.

## Supplemental information

Document S1. Supplemental methods, Figures S1–S41, and Tables S1–S8

Document S2. Article plus supplemental information

## Footnotes

It can be found online at https://doi.org/10.1016/j.xinn.2023.100445.
