# Supplementary material for: Physical regulation of copper catalyst with a hydrophobic promoter for enhancing CO2 hydrogenation to methanol
Source: Innovation (Camb). 2023 May 22;4(4):100445. doi: 10.1016/j.xinn.2023.100445 (PMC10251151; doi:10.1016/j.xinn.2023.100445)
Supplement: Document S1. Supplemental methods, Figures S1–S41, and Tables S1–S8 [file mmc1.pdf]

The Innovation, Volume *4*

Supplemental Information

Physical regulation of copper catalyst with a hydrophobic promoter for enhancing CO_2 hydrogenation to methanol

Hangjie Li, Wei Fang, Ling-Xiang Wang, Yifeng Liu, Lujie Liu, Tulai Sun, Ciqi Liao, Yihan Zhu, Liang Wang, and Feng-Shou Xiao

Experimental details

Materials

Tetraethylorthosilicate (TEOS, >99%), Diethoxydimethylsilane (DEMS), divinylbenzene (DVB), azobis isobutyronitrile (AIBN), zinc nitrate hexahydrate [$Zn(NO_3)_2 \cdot 6H_2O$], cerium nitrate hexahydrate [$Ce(NO_3)_3 \cdot 6H_2O$], lanthanum nitrate hexahydrate [$La(NO_3)_3 \cdot 6H_2O$], zirconium dioxide (ZrO_2), ethanol, anatase (TiO_2), and *n*-hexane (>99%, GC) were purchased from Aladdin Chemical Reagent Company. Ammonia solution ($NH_3 \cdot H_2O$, 28 *wt%*), copper nitrate trihydrate [$Cu(NO_3)_2 \cdot 3H_2O$], and quartz sand were obtained from Sinopharm Chemical Reagent Co. Ltd., polyacrylonitrile (PAN), polyamide (PA), polytetrafluoroethylene (PTFE) were provided from Adamas Reagent Ltd., silicalite-1 (S-1) was supported by Nankai university. γ-Al_2O_3 was obtained from Shanghai Macklin Biochemical Co. Ltd. All chemicals were used as received without further purification. Feed gas (containing $H_2/CO_2/Ar$, $H_2/CO/CO_2/Ar$, H_2/N_2, H_2/Ar, D_2/Ar, CO, *etc.*) was supplied by Hangzhou Jingong special gas Co. Ltd.

Catalyst preparation

Synthesis of SiO_2. As a typical run for the synthesis of SiO_2, 7.49 g of TEOS were dropped into a mixed liquor containing 80 mL of ethanol, 100 mL of water, and 6 mL of aqueous ammonia. After stirring at room temperature for 12 h, the liquor was removed by distillation under vacuum to get the solid powder, which was then dried at 100 °C overnight to obtain the amorphous silica (SiO_2).

Synthesis of Cu/SiO_2 catalyst. 12.05 g of $Cu(NO_3)_2 \cdot 3H_2O$ was dissolved in a mixed solution containing 300 mL of deionized water and 200 mL of ethanol. Then, 12 mL of 28% ammonia aqueous solution was then dropped into it. The mixed solution was stirred for 30 minutes to form a copper ammonia complex solution. 6 g of as-synthesized SiO_2 was subsequently added into the copper ammonia solution and stirred for 24 h. Then, the suspension was subsequently filtered and washed with diluent ammonia solution 5 times. The precursor was then dried at 100 °C for 10 h and calcined in air at 500 °C for 5 h to obtain the Cu/SiO_2 catalyst. The catalyst was pre-reduced with gaseous hydrogen before the CO_2 hydrogenation test.

Synthesis of Cu/SiO_2-Me catalyst. As a typical run for the synthesis of Cu/SiO_2-30Me. 0.7 g of Cu/SiO_2 catalyst was dispersed in a mixed liquor containing 40 mL of ethanol, 50 mL of water, and 3 mL of aqueous ammonia. Subsequently, 0.74 g of DEMS was dropped into the aforementioned solution. After stirring at room temperature for 34, the liquor was filtered and washed with deionized water for 5 times. The precursor was then dried at 100 °C for 10 h and calcined in air at 350 °C for 5 h to obtain the Cu/SiO_2-30Me catalyst. The catalyst was pre-reduced with gaseous hydrogen before the CO_2 hydrogenation test.

Synthesis of SiO_2-Me. As a typical run for the synthesis of SiO_2-10Me, 7.49 g of TEOS and 0.592 g of DEMS were dropped into a mixed liquor containing 80 mL of ethanol, 100 mL of water, and 6 mL of aqueous ammonia (28 wt%). After stirring at room temperature for 12 h, the liquor was removed by distillation under vacuum to get the solid powder, which was then dried at 100 °C overnight to obtain the amorphous silica modified with methyl groups (SiO_2-10Me). Similarly, the SiO_2-30Me sample was prepared following the same procedures by adjusting the amount of TEOS and DEMS at 5.824 g and 1.776 g, respectively.

Synthesis of Cu/SiO_2-Me(DL). Compared with Cu/SiO_2-Me, the Cu/SiO_2-Me(DL) catalysts were synthesized by firstly organic group modifying and then Cu loading. As a typical run for the synthesis of Cu/SiO_2-10Me(DL), 12.05 g of $Cu(NO_3)_2 \cdot 3H_2O$ was dissolved in a mixed solution containing 300 mL of deionized water and 200 mL of ethanol. Then, 12 mL of aqueous ammonia solution (28 wt%) was then dropped into it. The mixed solution was stirred for 30 minutes to form a copper ammonia complex solution. 6 g of as-synthesized SiO_2-10Me was subsequently added into the copper ammonia solution

and stirred for 24 h. Then, the suspension was subsequently filtered and washed with diluent ammonia solution 5 times. The precursor was dried at 100 °C for 10 h and calcined in air at 350 °C for 5 h to obtain the Cu/SiO_2-10Me(DL) catalyst. Similarly, the Cu/SiO_2-30Me(DL) sample was prepared from the same procedures by using SiO_2-30Me as support.

Synthesis of nonporous PDVB. 0.5 g of AIBN was added to 10 g of divinylbenzene under stirring at room temperature for 1 h, then the liquor was transferred into an autoclave and thermally treated at 100 °C for 24 h. The obtained solid was washed with methanol and dried at 100 °C overnight to obtain the nonporous polydivinylbenzene, which was denoted as PDVB.

Synthesis of CuZnAl and CuZnZr catalysts. As a typical run for the synthesis of CuZnAl, 4 mL aqueous solution containing 1.412 g $Cu(NO_3)_2\cdot 3H_2O$ and 0.571 g $Zn(NO_3)_2\cdot 6H_2O$ was dropwise added into 2.0 g of γ-Al_2O_3 powder and ZrO_2 powder under ultrasonic auxiliary for 60 min. After that, the precursor was dried at 100 °C for 12 h and calcined in air at 500 °C for 5 h to obtain the CuZnAl and CuZnZr catalysts.

Synthesis of Zn-Cu/SiO_2, Ce-Cu/SiO_2, and La-Cu/SiO_2 catalysts. 1 g of as-synthesized Cu/SiO_2 powder was dispersed in 100 mL of $Zn(NO_3)_2$, $Ce(NO_3)_3$, and $La(NO_3)_3$ solution (0.1 M). The suspension was stirred for 24 h at room temperature and then filtered and washed with deionized water for 5 times. The precursor was then dried at 100 °C for 10 h and calcined in air at 500 °C for 5 h.

Catalyst characterization

The textural parameters of the catalysts were determined by N_2 sorption isotherms using Micromeritics ASAP 2020 specific surface area and porous physical adsorption analyzer. Prior to the measurements, all samples were pretreated at 200 °C under vacuum for 10 h to remove the surface contaminants. Scanning electron microscopy (SEM) experiments were performed with Hitachi SU-8010 electron microscopes. IR characterization was performed with Thermo Nicolet NEXUS 470 FT-IR system. Thermogravimetric curves (TG) were performed on an SDT Q600 Simultaneous DSC-TGA in flowing air with a heating rate of 10 °C/min. TG-MS profiles were obtained from a METTLER TGA/DSC instrument connected to a mass spectrometer (LCD200M, TILON). ^{13}C NMR spectra were obtained from a Bruker Avance III HD 400 MHz spectrometer. Water-droplet contact angles were performed with Belsorp MaX II system at room temperature and humidity at ~25 °C and ~40 %. Water adsorption tests were performed with a video-based contact angle measuring device (OCA 20, Dataphysics Co. Ltd). The Cu dispersion and the temperature-programmed desorption of CO_2 (CO_2-TPD) for the catalysts were tested on BELCAT-T-SP apparatus equipped with a thermal conductivity detector (TCD). The compositions of the catalysts were detected by inductively coupled plasma optical emission spectrometry (ICP-OES) analysis (Perkin-Elmer 3300DV). The transmission electron microscopy (TEM) and high-resolution TEM (HRTEM) images were obtained from FEI Tecnai F20 microscope and FEI Spectra300. X-ray photoelectron spectroscopy (XPS) with Auger electron spectroscopy (AES) was conducted with a Thermo-Fischer ESCALAB 250Xi spectrometer. X-ray absorption fine structure (XAFS) spectroscopy at the Cu K-edge (E_0 = 8979 eV) was performed at the 1W1B beamline of the Beijing Synchrotron Radiation Facility (BSRF) operated at 2.5 GeV with a maximum electron current of about 250 mA.

Catalytic tests

The catalytic CO_2 hydrogenation was conducted in a continuous-flow fixed-bed reactor. As a typical run, 0.2 g of the as-prepared catalyst was fixed in the middle of the reactor. The catalyst was pre-reduced in 10 *vol%* H_2 flow (20 mL min^{-1}) at 300 °C for 8 h, then the gas was switched to a mixture of CO_2 and hydrogen at appropriate pressure (e.g. 3 MPa) and temperature (e.g. 210-240 °C) to start the reaction. The gaseous products were analyzed by two online gas chromatographs with a thermal conductivity detector (TCD, FULI 9790) and a flame ionization detector (FID, FULI 9790). The GHSV (mL g_{cat}^{-1} h^{-}

[1]) was calculated according to the weight of the Cu/SiO_2 catalyst amount in the reactor, and the PDVB promoter was not considered.

The CO_2 conversion, products selectivity, and the space-time yield (STY) of CH_3OH were calculated according to the following formulas:

$X_{(CO2)} = (n_{in(CO2)} - n_{out(CO2)})/n_{in(CO2)} \times 100\%$ ······(1)

$S_{(CO)} = n_{out(CO)} /(n_{in(CO2)} - n_{out(CO2)}) \times 100\%$ ······(2)

$S_{(MeOH)} = n_{out(MeOH)} /(n_{in(CO2)} - n_{out(CO2)}) \times 100\%$ ······(3)

$STY_{(MeOH)}=X_{(CO2)}*S_{(MeOH)}*M_{(MeOH,\ g/mol)}*n_{in(CO2,\ mol/h)}/m_{(Cu\ in\ catalyst,\ Kg)}$ ······(4)

Where n is the mole rate (mol/h) of CO_2 and products CO and MeOH.

***In-situ* FTIR**

In-situ DRIFTS of CO_2 hydrogenation over Cu/SiO_2, Cu/SiO_2-PDVB catalyst was performed at 240 °C. The spectra were collected using an FTIR spectrometer (Thermo, Nicolet 6700) equipped with an MCT detector. In a typical run for the water pre-treated Cu/SiO_2, 20 mg of Cu/SiO_2 catalyst was reduced by a 10 *vol%* H_2/Ar (20 mL min^{-1}) at 300 °C for 60 min followed and purged with a 20 mL min^{-1} 1.0 *vol*% H_2O/Ar (20 mL min^{-1}) at 240 °C for 4 h. After that, the background spectrum (32 scans) was obtained at 240 °C in Ar flow (20 mL min^{-1}). Subsequently, the *in-situ* DRIFT spectra were recorded during the CO_2 hydrogenation reaction (240 °C, ambient pressure, $H_2O/H_2/CO_2$/Ar at 72/24/4, *vol%*, 10 mL min^{-1}). For the test of Cu/SiO_2 catalyst without water pre-treatment, *In-situ* DRIFTS of CO_2 hydrogenation over normal Cu/SiO_2 was operated following similar procedures except without the water pretreatment step.

In-situ FTIR spectroscopy of hydroxide species on Cu/SiO_2, water pre-treated Cu/SiO_2 and Cu/SiO_2-PDVB samples was performed on a Micromeritics Perkin-Elmer Spectrum TM GX spectrometer. Prior to the scanning, all samples were heated at 200 °C in a vacuum system for 10 min to remove the adsorbed water.

Methanol decomposition test

The catalytic methanol decomposition test was conducted in a continuous-flow fixed-bed reactor. As a typical run, 100 mg of catalyst was pre-reduced by 10 *vol%* H_2 with a flow rate of 30 mL min^{-1} at 300 °C for 60 min. After cooling to 240 °C, the methanol feed (7.4%CH_3OH/0.9CH_4/91.7%N_2, or 4.2%CH_3OH/1.8%H_2O/0.9CH_4/93.1%N_2, 30 mL min^{-1}) was purged into the reactor, and the gaseous products were analyzed by a gas chromatograph with a flame ionization detector (FID, FULI 9790).

CO-adsorption test

The CO-adsorption test was performed on an FTIR spectrometer (Thermo, Nicolet 6700) equipped with an MCT detector. The samples were reduced under 10% H_2/90% Ar (30 mL min^{-1}) at 300 °C for 0.5 h and then purged by Ar (30 mL min^{-1}) under cooling to 25 °C. Subsequently, the temperature was further reduced to −100 °C with liquid nitrogen. The background spectrum (64 scans) was obtained at −100 °C in Ar flow (30 mL min^{-1}). After that, CO gas flow (10 mL min^{-1}) was purged into the reactor for 30 min. After saturated adsorption, the FT-IR spectra were recorded with the purging of Ar flow of 30 mL min^{-1}.

H/D exchange

H/D exchange was carried out in a fixed-bed quartz reactor with a mass spectrum instrument (SRD200M, TILON GRP TECHNOLOGY LIMITED). 20 mg of catalyst was pre-reduced by 10 *vol%* H_2 with a flow rate of 30 mL min^{-1} at 300 °C for 60 min. After cooling to 240 °C, the H_2 and D_2 were introduced, and the HD (m/z at 3) signals were collected.

TPSR test

Temperature-programmed surface reaction (TPSR) test with methanol feed was carried out in a fixed-bed quartz reactor with a mass spectrum instrument (SRD200M, TILON GRP TECHNOLOGY

LIMITED). 20 mg of catalyst was pre-reduced by 10 *vol%* H_2 with a flow rate of 30 mL min^{-1} at 300 °C for 60 min. After cooling to 50 °C, the methanol feed (1.0 *vol%* in Ar, 15 mL min^{-1}) was purged into the reactor, then the temperature was subsequently raised to 400 °C with a heating rate of 10 °C min^{-1}. The signals of methanol, CO, methane, and CO_2 in the emission gas were collected by a mass spectrometer.

Table S1. Data showing the catalytic performance of Cu/SiO_2-PDVB with different ratios of Cu/SiO_2 and PDVB in catalyzing CO_2 hydrogenation to methanol.[a]

Catalyst	CO_2 Conv. (%)	Selectivity (%)			Productivity
		CO	CH_4	MeOH	(g_{MeOH} Kg_{Cu}^{-1} h^{-1})
Cu/SiO_2-PDVB-1.0	10.2	52.5	1.0	46.5	558
Cu/SiO_2-PDVB-1.5	10.3	53.8	1.2	45.0	545
Cu/SiO_2-PDVB-2.0	10.3	54.1	1.1	44.8	543

[a] Reaction conditions: 240 °C, 3 MPa, SV of 6000 mL g_{cat}^{-1} h^{-1}, H_2/CO_2/Ar ratio at 72/24/4 *vol%*.

Note: We have studied the CO_2 hydrogenation over Cu/SiO_2-PDVB with weight ratios of PDVB to Cu/SiO_2 at 1.5 and 2.0 (Cu/SiO_2-PDVB-1.5 and Cu/SiO_2-PDVB-2.0). As shown in Table S1, the Cu/SiO_2-PDVB-1.0, Cu/SiO_2-PDVB-1.5, and Cu/SiO_2-PDVB-2.0 catalysts gave very similar CO_2 conversion at 10.2~10.3 % and methanol selectivity at 44.8~46.5 %, respectively, giving similar methanol productivity of 543~550 g_{MeOH} Kg_{Cu}^{-1} h^{-1}. Therefore, the Cu/SiO_2-PDVB with a weight ratio of 1.0 was an optimum ratio in this work.

Table S2. Data showing the catalytic performance of Cu/SiO_2 and Cu/SiO_2-PDVB in CO_2 hydrogenation.[a]

Entry	Catalyst	Temp. (°C)	SV (mL g_{cat}^{-1} h^{-1})	CO_2 Conv. (%)	Selectivity (%)			Productivity (g_{MeOH} Kg_{Cu}^{-1} h^{-1})
					CO	CH_4	MeOH	
1	Cu/SiO_2	210	18000	1.8	9.3	0	90.7	576
2	Cu/SiO_2	220	18000	3.1	12.5	0	87.5	956
3	Cu/SiO_2	230	18000	4.1	16.0	0	84.0	1214
4	Cu/SiO_2	240	18000	4.8	18.8	0	81.2	1403
5	Cu/SiO_2	210	6000	3.2	17.0	0	83.0	316
6	Cu/SiO_2	220	6000	4.3	25.0	0	75.0	379
7	Cu/SiO_2	230	6000	5.2	32.0	0	68.0	416
5	Cu/SiO_2	240	3000	8.1	49.0	0	51.0	243
6	Cu/SiO_2	240	6000	5.9	38.7	0	61.3	420
7	Cu/SiO_2	240	9000	5.1	35.0	0	65.0	585
8	Cu/SiO_2	240	12000	4.9	25.0	0	75.0	864
9	Cu/SiO_2	240	24000	4.5	17.0	0	83.0	1756
10	Cu/SiO_2-PDVB [b]	210	18000	4.0	14.0	0	86.0	1213
11	Cu/SiO_2-PDVB	220	18000	4.4	17.8	0.1	82.1	1272
12	Cu/SiO_2-PDVB	230	18000	5.9	30.0	0.1	69.9	1454
13	Cu/SiO_2-PDVB	240	18000	6.8	33.0	0.2	66.8	1602
14	Cu/SiO_2-PDVB	240	6000	10.2	52.5	1.0	46.5	558
15	Cu/SiO_2-PDVB	210	6000	5.4	25.6	0	74.4	472
16	Cu/SiO_2-PDVB	220	6000	6.4	34.8	0.2	65.0	490
17	Cu/SiO_2-PDVB	230	6000	8.1	45.4	0.5	54.1	519
18	Cu/SiO_2-PDVB	240	3000	12.1	57.3	1.5	41.2	293
19	Cu/SiO_2-PDVB	240	9000	9.1	47.5	0.5	52.0	834
20	Cu/SiO_2-PDVB	240	12000	7.9	41.8	0.2	58.0	1077
21	Cu/SiO_2-PDVB	240	24000	6.2	28.0	0	72.0	2099

[a] Reaction conditions: 3 MPa, a feed gas of $H_2/CO_2/Ar$ at 72/24/4 *vol%*, the SV (mL g_{cat}^{-1} h^{-1}) was calculated according to the weight of Cu/SiO_2 catalyst amount in the reactor, and the PDVB promoter was not considered. The SV was adjusted by changing the flow rate of feed gas ($H_2/CO_2/Ar$) with a constant Cu/SiO_2 amount of 0.2 g for both Cu/SiO_2 (20-40 mesh) and Cu/SiO_2-PDVB catalysts; [b] Cu/SiO_2-PDVB represents the catalyst with powder mixing manner (the Cu/SiO_2 powder was mixed with an equivalent weight of PDVB powder, and then squeezed and crushed into granules with 20-40 mesh size for tests).

Note: Table S2 summarized the catalytic data of Cu/SiO_2 and Cu/SiO_2-PDVB catalysts at different conditions. At 210 ºC with a feeding rate at 18000 mL g_{cat}^{-1} h^{-1}, the CO_2 conversion and methanol selectivity were 4.0% and 86.0% over Cu/SiO_2-PDVB, resulting in the methanol productivity of 1213 g_{MeOH} Kg_{Cu}^{-1} h^{-1}. At 240 ºC with a feed rate at 6000 mL g_{cat}^{-1} h^{-1}, the CO_2 conversion and methanol selectivity were 10.2% and 46.5% over Cu/SiO_2-PDVB, giving a methanol productivity of 558 g_{MeOH} Kg_{Cu}^{-1} h^{-1}. These employed reaction conditions lead to the high methanol productivity at 210 ºC than that at 240 ºC.

Under the same temperature and gas feeding rate, the PDVB also obviously enhanced the CO_2 conversions (Table S2). Although the methanol selectivity was reduced by improving the CO_2 conversion by PDVB, the methanol productivity was always improved. For example, at 210 ºC with a feed rate of 18000 mL g_{cat}^{-1} h^{-1}, the methanol productivities were 576 g_{MeOH} Kg_{Cu}^{-1} h^{-1} over Cu/SiO_2 catalyst (CO_2 conversion at 1.8%, methanol selectivity at 90.7%), which was enhanced to 1213 g_{MeOH} Kg_{Cu}^{-1} h^{-1} over Cu/SiO_2-PDVB catalyst (CO_2 conversion at 4.0%, methanol selectivity at 86.0%). At 230 ºC with a feed

rate at 18000 mL g_{cat}^{-1} h^{-1}, the methanol productivities were 1214 and 1454 g_{MeOH} Kg_{Cu}^{-1} h^{-1} over Cu/SiO_2 and Cu/SiO_2-PDVB catalysts, respectively. At 230 ºC with a feed rate of 6000 mL g_{cat}^{-1} h^{-1}, the methanol productivities were 416 and 519 g_{MeOH} Kg_{Cu}^{-1} h^{-1} over Cu/SiO_2 and Cu/SiO_2-PDVB catalysts, respectively.

Table S3. Data showing the catalytic performance of difference catalysts in CO_2 hydrogenation to MeOH.[a]

Catalyst	CO_2 Conv. (%)	Selectivity (%)		
		CO	CH_4	MeOH
Cu/SiO_2	5.9	38.7	0	61.3
Cu/SiO_2-10Me(DL)	7.8	47.0	0.5	52.5
Cu/SiO_2-30Me(DL)	8.7	53.0	1.9	45.1
Cu/SiO_2-PDVB [b]	10.2	52.5	1.0	46.5

[a] Reaction conditions: 240 °C, 3 MPa, SV of 6000 mL g_{cat}^{-1} h^{-1}, a feed gas of H_2/CO_2/Ar at 72/24/4 *vol%*, the SV (mL g_{cat}^{-1} h^{-1}) was calculated according to the weight of Cu/SiO_2 catalyst amount in the reactor, the quartz sand and PDVB promoter were not considered. [b] Cu/SiO_2-PDVB represents the catalyst in a powder mixing manner (the Cu/SiO_2 powder was mixed with an equivalent weight of PDVB powder, and then squeezed and crushed into granules with 20-40 mesh size for tests).

Note: We also prepared another series of samples for comparison by organosilane modification before Cu loading. We directly loaded Cu nanoparticles on the silica support with ~10% and ~30% fraction of Si modified with methyl groups (co-hydrolysis of tetraethoxysilane and methylsilane), obtaining the Cu/SiO_2-10Me(DL) and Cu/SiO_2-30Me(DL) catalysts. As shown in Table S3, the Cu/SiO_2-10Me(DL) and Cu/SiO_2-30Me(DL) exhibited CO_2 conversions at 7.8 and 8.7% and methanol selectivities at 52.5% and 45.1%. Such performances are higher than that of the Cu/SiO_2 catalyst without hydrophobic group modification, confirming the importance of catalyst hydrophobicity. However, such performance, which relies on the chemical modification that changed the surface structure, was still lower than that of the Cu/SiO_2-PDVB catalyst.

Table S4. Data showing the gas composition in effluent from CO_2 hydrogenation over Cu/SiO_2-PDVB catalyst.

Feed gas	Concentration (mol%)	Effluent gas	Concentration(mol%)
H_2	72.00	H_2	68.79
CO_2	24.00	CO_2	22.08
Ar	4.00	Ar	4.10
-	-	CO	1.30
-	-	MeOH	1.17
-	-	CH_4	0.04
-	-	H_2O	2.52

[a] Reaction conditions: 240 °C, 3 MPa, SV of 6000 mL g_{cat}^{-1} h^{-1}, a feed gas of H_2/CO_2/Ar at 72/24/4 *vol%*. Cu/SiO_2-PDVB represents the catalyst in a powder mixing manner (the Cu/SiO_2 powder was mixed with an equivalent weight of PDVB powder, and then squeezed and crushed into granules with 20-40 mesh size for tests).

Table S5. Data showing the catalytic performance of CuZnAl-quartz sand, CuZnAl-PDVB, CuZnZr-quartz sand, and CuZnZr-PDVB in catalyzing CO_2 hydrogenation to methanol. [a]

Catalyst	CO_2 Conv. (%)	Selectivity (%)			Productivity
		CO	CH_4	MeOH	(g_{MeOH} Kg_{Cu}^{-1} h^{-1})
CuZnAl-quartz sand [b]	6.7	44.8	0	55.2	464
CuZnAl-PDVB [c]	9.1	50.4	0.6	49.0	559
CuZnZr-quartz sand	5.3	7.8	0	92.2	613
CuZnZr-PDVB [b]	7.2	12.6	5.5	81.9	739

[a] Reaction conditions: 3 MPa, 240 °C, SV of 6000 mL g_{cat}^{-1}, a feed gas of H_2/CO_2/Ar ratio at 72/24/4 *vol%*, and the SV (mL g_{cat}^{-1} h^{-1}) was calculated according to the weight of CuZnAl and CuZnZr catalyst in the reactor, where the quartz sand and PDVB promoter were not considered. [b] The CuZnAl and CuZnZr granules (0.2 g, 20~40 mesh) were diluted with quartz sand granules (0.1 g, 20-40 mesh). [c] CuZnAl-PDVB and CuZnZr represent the catalysts in a powder mixing manner (the CuZnAl and CuZnZr powders were mixed with an equivalent weight of PDVB powder, and then squeezed and crushed into granules with 20-40 mesh size for tests). The CuZnAl-quartz sand and CuZnZr-quartz sand have the same volume as CuZnAl-PDVB and CuZnZr-PDVB in the catalyst bed.

Note: We also studied the effect of PDVB in promoting CO_2 hydrogenation over other catalysts. To investigate the effect of PDVB, the CO_2 conversions were controlled to lower than the thermodynamic equilibrium. The bare CuZnAl and CuZnZr catalysts exhibited CO_2 conversion at 6.7 and 5.3% and methanol selectivity at 55.2 and 92.2%, which are similar to the results reported Cu-based catalysts with similar Cu contents.[1] After mixing with PDVB, the CO_2 conversion was obviously enhanced to 9.1 and 7.2% with a slightly decreased methanol selectivity at 49.0 and 81.9% for CuZnAl-PDVB and CuZnZr-PDVB catalysts. As a result, the methanol productivity was increased from 464 to 559 g_{MeOH} Kg_{Cu}^{-1} h^{-1} for CuZnAl after mixing with PDVB, and from 613 to 739 g_{MeOH} Kg_{Cu}^{-1} h^{-1} for CuZnZr after mixing with PDVB.

Table S6. Data showing the catalytic performance of various catalysts in CO_2 hydrogenation to methanol.[a]

Catalyst	CO_2 Conv. (%)	Selectivity (%)			Productivity (g_{MeOH} Kg_{Cu}^{-1} h^{-1})
		CO	CH_4	MeOH	
$CuZn/SiO_2$	6.7	43.5	0	56.5	436
$CuZn/SiO_2$-PDVB [b]	11.2	60.0	2.5	37.5	494
$CuCe/SiO_2$	6.7	44.1	0	55.9	440
$CuCe/SiO_2$-PDVB	11.0	61.9	0.6	37.5	485
$CuLa/SiO_2$	6.9	45.1	0	54.9	445
$CuLa/SiO_2$-PDVB	13.7	61.2	0.6	38.2	615

[a] Reaction conditions: 3 MPa, 240 °C, SV of 6000 mL g_{cat}^{-1}, and a feed gas of H_2/CO_2/Ar ratio at 72/24/4 *vol%*.

[b] $CuZn/SiO_2$-PDVB, $CuCe/SiO_2$-PDVB, and $CuLa/SiO_2$-PDVB represent the catalyst in the powder mixing manner (the $CuZn/SiO_2$, $CuCe/SiO_2$, and $CuLa/SiO_2$ powders were mixed with an equivalent weight of PDVB powder, and then squeezed and crushed into granules with 20-40 mesh size for tests).

Note: We have studied the CO_2 hydrogenation over the $CuZn/SiO_2$, $CuCe/SiO_2$, and $CuLa/SiO_2$ catalysts. As shown in Table S6, the bare $CuZn/SiO_2$, $CuCe/SiO_2$, and $CuLa/SiO_2$ catalysts exhibited CO_2 conversion at 6.7, 6.7, and 6.9 % and methanol selectivity at 56.5, 55.9, and 54.9%, respectively. After mixing with PDVB, the CO_2 conversions were obviously increased to 11.2, 11.0, and 13.7% with slightly decreased methanol selectivities at 37.5, 37.5, and 38.2% for $CuZn/SiO_2$-PDVB, $CuCe/SiO_2$-PDVB, and $CuLa/SiO_2$-PDVB catalysts. These results confirm the promotion effect of PDVB for the CO_2 hydrogenation.

Table S7. Data showing the catalytic performance of CO_2 hydrogenation to methanol with/without the addition of CO. [a]

Catalyst	CO_2 Conv. (%)	Selectivity (%)			Productivity
		CO	CH_4	MeOH	(g_{MeOH} Kg_{Cu}^{-1} h^{-1})
Cu/SiO_2-PDVB [b]	10.2	52.5	1.0	46.5	558
Cu/SiO_2-PDVB [c]	8.8	40.2	1.0	58.8	608
CuLa/SiO_2-PDVB [b]	13.7	61.2	0.6	38.2	615
CuLa/SiO_2-PDVB [c]	10.9	48.2	0.5	51.3	657

[a] Reaction conditions: 240 °C, 3 MPa, and SV of 6000 mL g_{cat}^{-1} h^{-1}. Cu/SiO_2-PDVB and CuLa/SiO_2-PDVB represent the catalysts in the powder mixing manner (the Cu/SiO_2 and CuLa/SiO_2 powders were mixed with an equivalent weight of PDVB powder, and then squeezed and crushed into granules with 20-40 mesh size for tests).

[b] Using a feed gas of H_2/CO_2/Ar ratio at 72/24/4 *vol%* (CO-free)

[c] Using a feed gas of H_2/CO_2/CO/Ar ratio at 72/21/4/3 *vol%* (CO-added).

Note: We have studied the CO_2 hydrogenation over Cu/SiO_2-PDVB and CuLa/SiO_2-PDVB catalysts using a feeding gas with a small content of CO (H_2/CO_2/CO/Ar ratio at 72/21/4/3, vol%). As shown in Table S7, in the absence of CO, the Cu/SiO_2-PDVB and CuLa/SiO_2-PDVB catalysts exhibited CO_2 conversion at 10.2 and 13.7% and CO selectivity at 52.5 and 61.2 %, respectively. After introduction of CO into the feed gas, the CO selectivity was suppressed to 40.2 and 48.2% with slightly decreased CO_2 conversion at 8.8 and 10.9 %, respectively. As a result, the methanol productivity was increased from 558 and 615 to 608 and 657 g_{MeOH} Kg_{Cu}^{-1} h^{-1} over Cu/SiO_2-PDVB and CuLa/SiO_2-PDVB catalysts by adding CO in the feed. This trend is similar to that reported previously. [2,3]

Table S8. Data showing the performances of selected catalysts with comparable Cu contents for CO_2 hydrogenation to methanol under similar reaction conditions.

Catalysts	Temp. (°C)	P (MPa)	SV (mL g^{-1} h^{-1})	CO_2 conv. (%)	Selectivity (%)		Ref.
					CO	MeOH	
Cu@UiO-bpy	250	4	1600	5.6	48.1	51.9	1
CZAZ-20Al	220	3	2000	8.6	46.2	53.8	4
LCZ-173	250	5	3600	6.4	39.5	57.9	5
LYCZ-8273	250	5	3600	5.0	37.0	55.7	5
S-CZZ-500	240	3	3600	9.3	41.1	58.9	6
CZZ-600	240	3	3600	8.1	61.4	38.6	6
Cu-ZnO-Al_2O_3	250	3	2600	6.3	31.4	68.6	7
CuZnCeO_x	220	2	2400	8.2	44.0	56.0	8
Cu/SiO_2	230	2.5	-	<3.5	51.0	49.0	9
Cu/HAl	240	3.0	7200	5.6	58.3	41.7	10
Cu/UAl	240	3.0	7200	6.4	64.3	35.7	10
Cu@m-SiO_2	250	5	6000	9.8	73.5	26.5	11
Cu/SiO_2-AE	260	3	16000	8.2	59.8	40.2	3
AE-Cu/SiO_2	230	3	2060	~6.0	-	~32.0	12
Cu/SiO_2	250	4.1	3600	2.8	-	15	13
CS25	260	2	10000	5.9	72.3	27.5	14
CuSi-NT	240	3	12000	~5.8	-	~55.0	15
Cu/SiO_2	250	3	12000	3.5	-	37.5	16
CuZnSi-AEM	240	2	4000	<5.0	~43.0	~54.0	17
Cu/SiO_2-PDVB	240	3	6000	10.2	52.5	46.5	This work

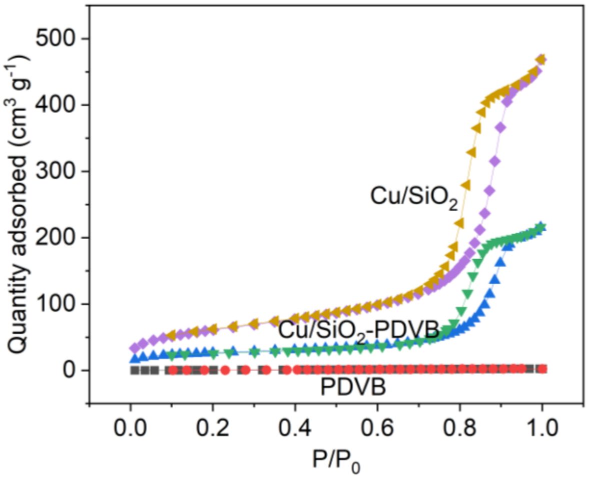

Figure S1. Nitrogen sorption isotherms of as-synthesized Cu/SiO_2, Cu/SiO_2-PDVB, and PDVB samples.

Note: The non-porous PDVB with almost undetectable N_2 sorption (BET surface area < 5 m^2/g) was employed in this work, which could not change the position of the hysteresis loop in the isotherms of the Cu/SiO_2 sample. As shown in Figure S1, the Cu/SiO_2-PDVB adsorbed less nitrogen but gave a similar position of hysteresis loops compared with Cu/SiO_2.

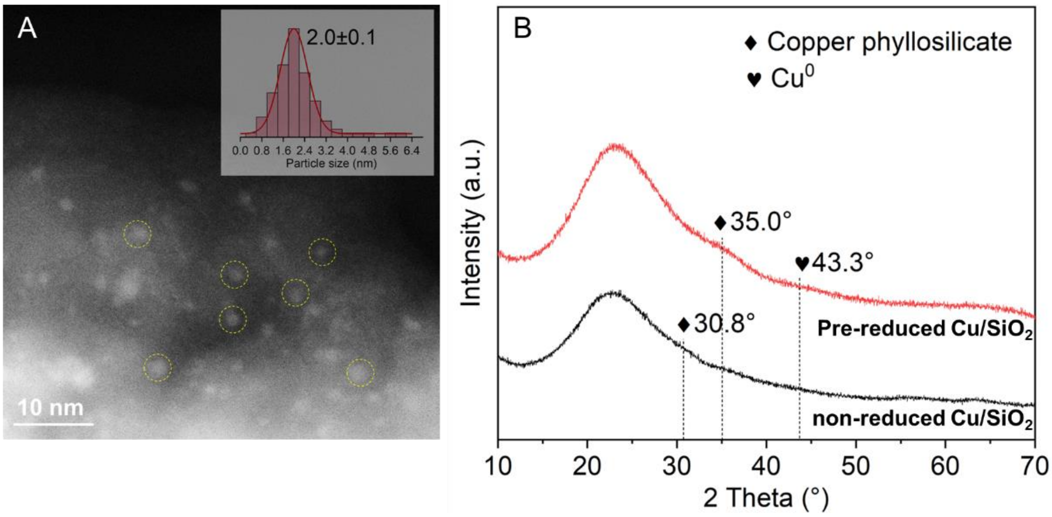

Figure S2. (A) STEM image showing the pre-reduced Cu/SiO_2. The copper nanoparticles were partially highlighted by yellow circles. (B) XRD patterns of the non-reduced and pre-reduced Cu/SiO_2 samples.

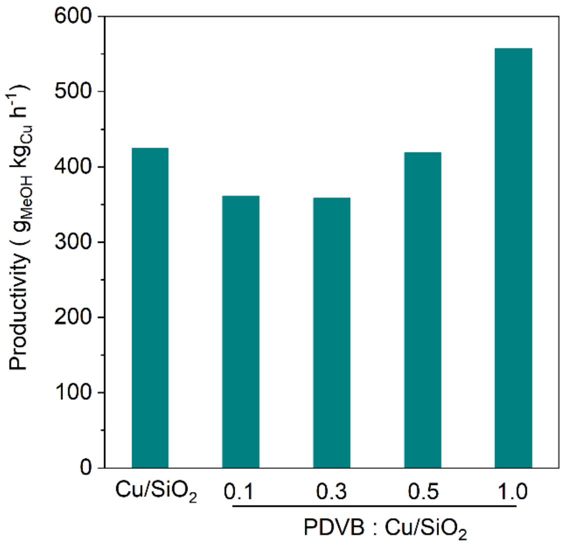

Figure S3. MeOH productivity in CO_2 hydrogenation over Cu/SiO_2 and Cu/SiO_2-PDVB catalysts with different weight ratios of Cu/SiO_2 and PDVB. Reaction conditions: 3 MPa, 240 °C, SV of 6000 mL g_{cat}^{-1} h^{-1}, a feed gas of H_2/CO_2/Ar at 72/24/4 *vol%*.

Note: The methanol productivities were similar over the catalysts with mixtures of 0.1~0.5 PDVB: Cu/SiO_2 (weight ratio) in the reaction at 240 °C, but the conversions and selectivities were obviously changed. Data showing the CO_2 conversions and methanol selectivities as a function of PDVB content are summarized in Figure S3. With raising the PDVB content from 0 to 0.5, the CO_2 conversions continuously increased from 5.9% to 9.5%, suggesting the promotion effect of PDVB for the Cu/SiO_2 catalyst. Simultaneously, the methanol selectivities were reduced. Because the methanol productivities were determined by CO_2 conversion and methanol selectivity, the methanol productivities were similar in these cases as an apparent result.

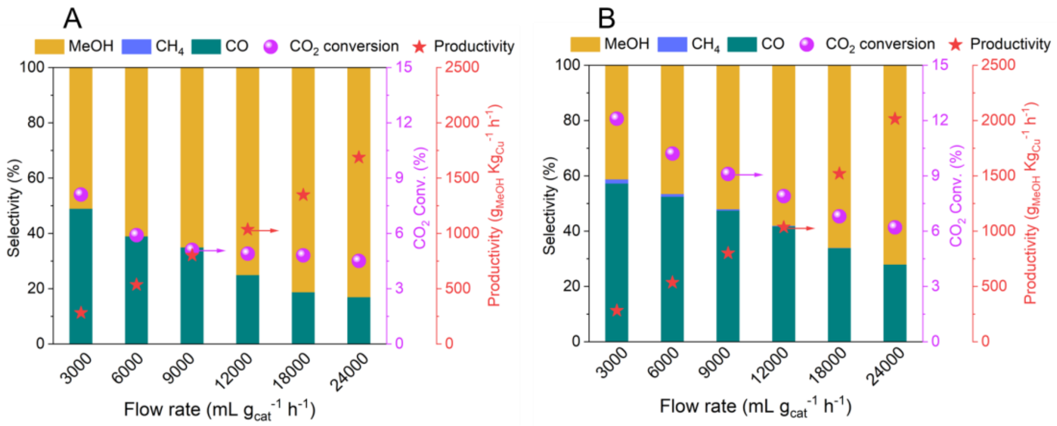

Figure S4. Data showing the CO_2 conversion, selectivity, and methanol productivity of (A) Cu/SiO_2 and (B) Cu/SiO_2-PDVB catalysts in CO_2 hydrogenation under varied flow rate at 3000-24000 mL g_{cat}^{-1} h^{-1}. Reaction conditions: 240 °C, 3 MPa, a feed gas of H_2/CO_2/Ar ratio at 72/24/4 *vol%*, the SV (mL g_{cat}^{-1} h^{-1}) was calculated according to the weight of Cu/SiO_2 catalyst in the reactor, the PDVB promoter was not considered. The SV was regulated by changing the flow rate of feed gas (H_2/CO_2/Ar) with a fixed Cu/SiO_2 amount of 0.2 g for both Cu/SiO_2 (20-40 mesh) and Cu/SiO_2-PDVB catalysts; [b] Cu/SiO_2-PDVB represents the catalyst with powder mixing manner (the Cu/SiO_2 powder was mixed with an equivalent weight of PDVB powder, and then squeezed and crushed into granules with 20-40 mesh size for tests).

Note: We also studied the effects of space velocity on the performances of Cu/SiO_2 and Cu/SiO_2-PDVB individually. As shown in Figure S4A, at 240 °C over Cu/SiO_2 catalyst, the CO_2 conversions were reduced by raising the gas feeding rates, giving 8.1%, 5.9%, 5.1%, 4.9%, 4.8%, and 4.5% under gas feeding rates at 3000, 6000, 9000, 12000, 18000, and 24000 mL g_{cat}^{-1} h^{-1}, respectively. Simultaneously, the methanol selectivities were continuously improved from 51.0% to 83.0%. At 240 °C over Cu/SiO_2-PDVB catalyst, a similar trend was observed (Figure S4B). These data confirm that longer residence time benefits enhancing the CO_2 conversion and reducing the methanol selectivity on both Cu/SiO_2 and Cu/SiO_2-PDVB catalysts, which is consistent with the general phenomenon in CO_2 hydrogenation to methanol.[18,19]

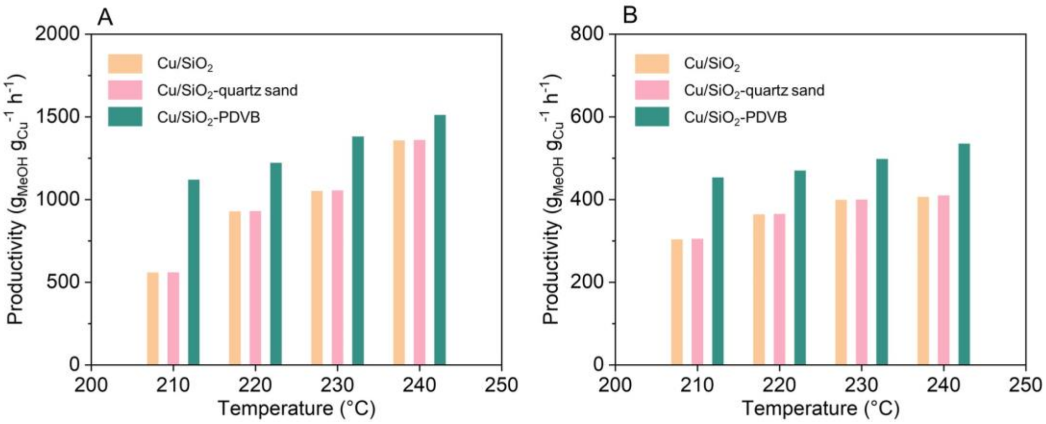

Figure S5. Data showing the methanol productivity of Cu/SiO_2, Cu/SiO_2-quartz sand, and Cu/SiO_2-PDVB catalysts in CO_2 hydrogenation under SV of (A) 18000 and (B) 6000 mL g_{cat}^{-1} h^{-1}. Reaction condition: 3 MPa, 210~240 °C, SV of 6000-18000 mL g_{cat}^{-1} h^{-1}, a feed gas of H_2/CO_2/Ar ratio at 72/24/4 *vol%*. The SV (mL g_{cat}^{-1} h^{-1}) was calculated according to the weight of the Cu/SiO_2 catalyst (20-40 mesh) in the reactor, and the PDVB promoter and inert quartz sand were not considered. Cu/SiO_2-quartz represents a mixture of Cu/SiO_2 granules (0.2 g, 20-40 mesh) and quartz sand granules (0.1 g, 20-40 mesh). Cu/SiO_2-PDVB represents the catalyst in a powder mixing manner (the Cu/SiO_2 powder was mixed with an equivalent weight of PDVB powder, and then squeezed and crushed into granules with 20-40 mesh size for tests). It should be noted that the Cu/SiO_2-quartz sand showed the same volume as Cu/SiO_2-PDVB.

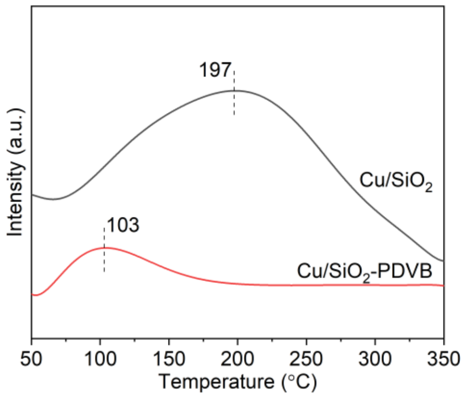

Figure S6. H_2O-TPD profiles of Cu/SiO_2 and Cu/SiO_2-PDVB.

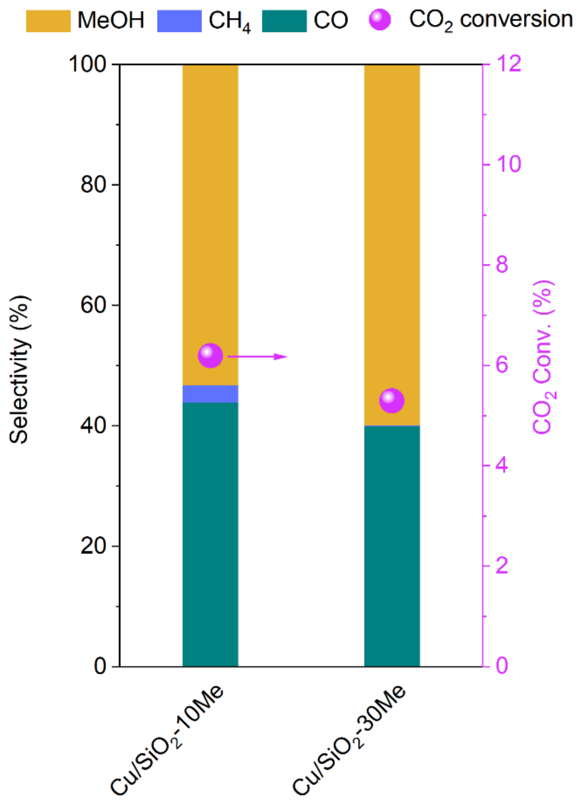

Figure S7. Catalytic performance of Cu/SiO_2-10Me and Cu/SiO_2-30Me catalysts in CO_2 hydrogenation. Reaction conditions: 3 MPa, 240 °C, SV of 6000 mL g_{cat}^{-1} h^{-1}, a feed gas of H_2/CO_2/Ar at 72/24/4 *vol%*.

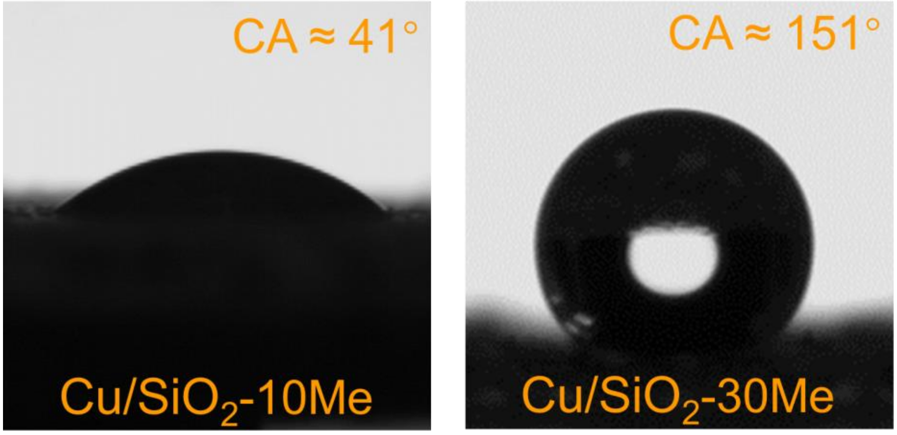

Figure S8. Water-droplet contact angles of the Cu/SiO_2-10Me and Cu/SiO_2-30Me catalysts.

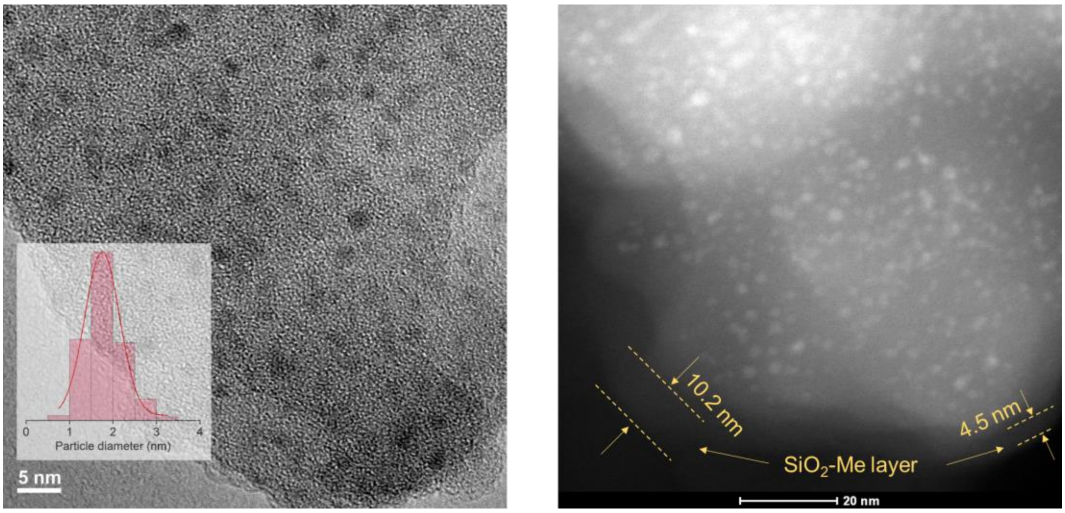

Figure S9. TEM images of the as-synthesized Cu/SiO_2-30Me catalyst.

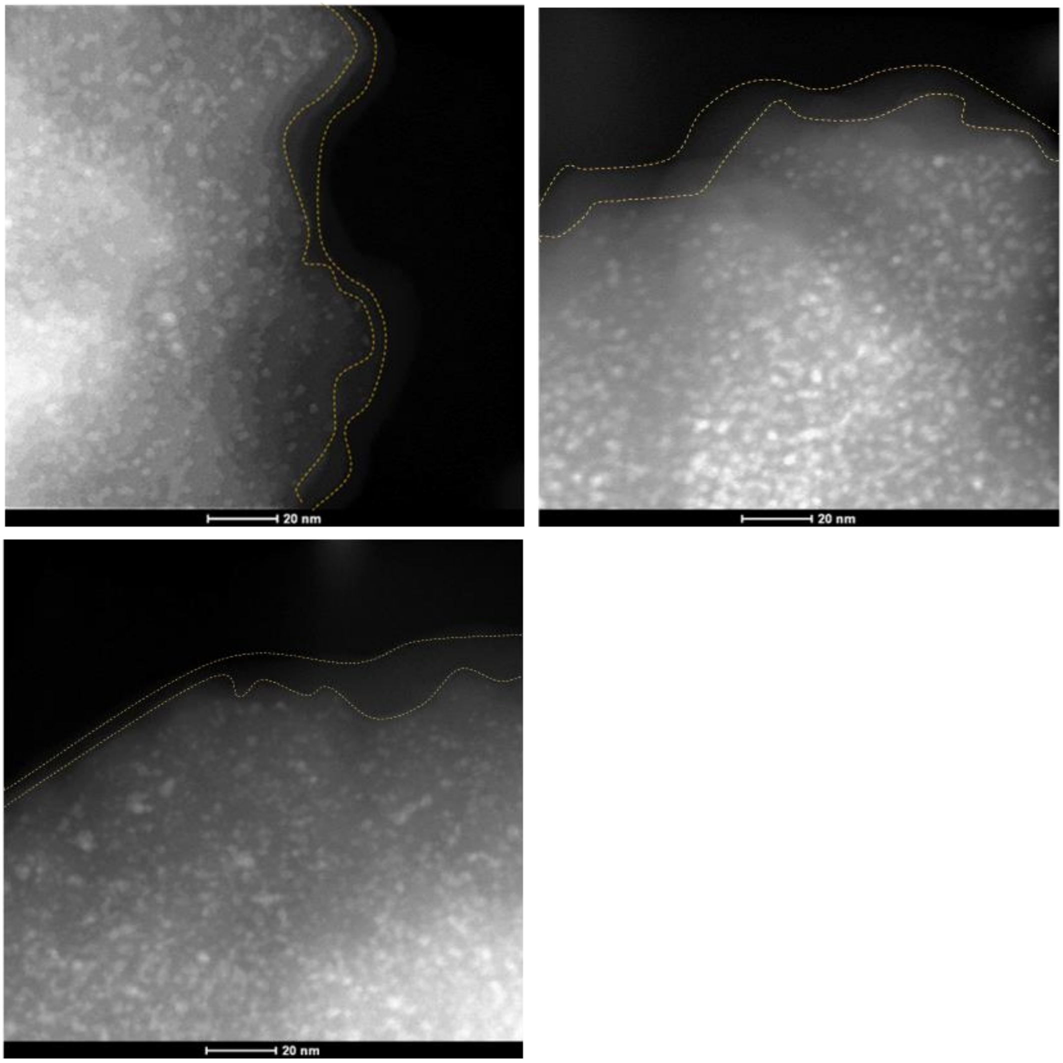

Figure S10. TEM images characterizing the different regions on Cu/SiO_2-30Me catalyst. The yellow curves highlight the silica sheath without Cu nanoparticles.

Note: The TEM images have provided clear observation of an amorphous layer, with slighter contrast relative to the silica matrix, on the Cu/SiO_2-30Me sample. A similar phenomenon has been observed in other different regions in this sample, giving similar amorphous layers (Figure S10). The Cu nanoparticles were all within the internal region of the silica areas, with almost undetectable ones on the external region of the support, which is also a typical feature of the encapsulated structures.[20,21]

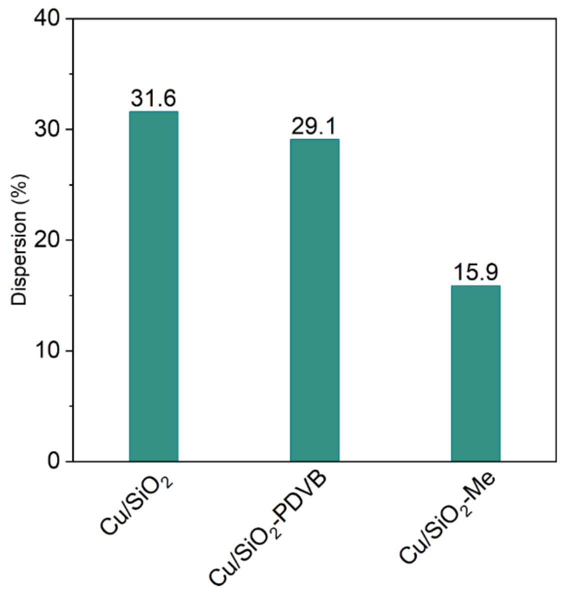

Figure S11. Cu dispersion of various catalysts. The error bounds were ±3%.

Note: The difference between the physical and chemical modulation methods was quantified by measuring the access of Cu surface sites to the adsorbate. By the N_2O-adsorption tests, the molar ratio of accessible Cu sites to the total amount of Cu species (Cu dispersion) were 31.6%, 29.1%, and 15.9% for the Cu/SiO_2, Cu/SiO_2-PDVB, and Cu/SiO_2-30Me catalysts, respectively (Figure S11). Similar Cu dispersion of the Cu/SiO_2-PDVB to that of the unmodified Cu/SiO_2 indicates the fully exposed Cu sites by the PDVB regulation method. In contrast, the much lower Cu dispersion of the Cu/SiO_2-Me catalyst is due to the blockage of the Cu surface by the chemical modification that led to the partial loss of active sites, in good agreement with those observed from the TEM results.

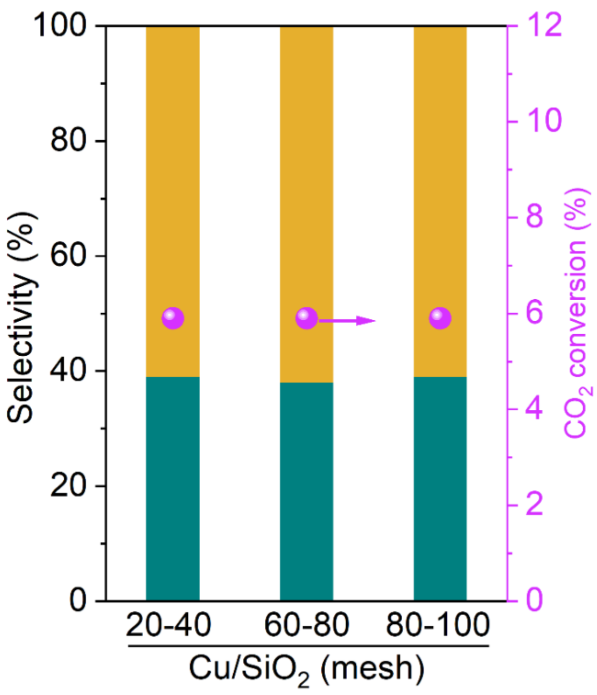

Figure S12. Data showing the catalytic performance of the Cu/SiO_2 catalyst with different granule sizes in CO_2 hydrogenation. Reaction conditions: 3 MPa, 240 °C, SV of 6000 mL g_{cat}^{-1} h^{-1}, a feed gas of H_2/CO_2/Ar ratio at 72/24/4 *vol%*.

Note: We used SV (mL g_{cat}^{-1} h^{-1}, based on the weight of Cu/SiO_2 component for Cu/SiO_2-PDVB catalyst, and the PDVB was not included for calculating the SV) in the manuscript to describe the reaction conditions. In addition, we also studied the F/V (mL mL_{cat}^{-1} h^{-1}, based on the volume of Cu/SiO_2 component for Cu/SiO_2-PDVB catalyst, and the PDVB was not included).[22]

As shown in Figure S12, the Cu/SiO_2 catalysts with granule sizes at 20-40, 60-80, and 80-100 mesh were used for the reaction at 240 ºC with a gas feeding rate at 6000 mL g_{cat}^{-1} h^{-1}. In these cases, the F/V were very similar at 2083, 2273, and 2326 mL mL_{cat}^{-1} h^{-1} according to the packing densities of the catalyst with different granule sizes (2.88 cm^3/g for 20-40 mesh, 2.64 cm^3/g for 60-80 mesh, 2.58 cm^3/g for 80-100 mesh). We found that the CO_2 conversion (~5.9%) and methanol selectivity (~61.3%) were very similar in these cases. For the Cu/SiO_2-PDVB catalysts with granule sizes at 20~40, 60~80, and 80~100 mesh, the granule sizes influenced the catalysis over Cu/SiO_2-PDVB catalysts (mixture of Cu/SiO_2 granules with PDVB granules) more obviously, which is different from the phenomenon on Cu/SiO_2 catalyst (Figure 3A).

In sum, these results demonstrate that the CO_2 conversion and methanol selectivity could not be obviously influenced by the diluter-caused (e.g. quartz sand) different F/V values in the studied cases. These results are also supported by the phenomenon observed previously.[23] For the Cu/SiO_2 granules mixed with PDVB granules (20-40 mesh), the CO_2 conversion was obviously enhanced relative to the Cu/SiO_2 granules mixed with quartz sands with the same F/V, which should be reasonably due to the different wettability of the PDVB and quartz sands.

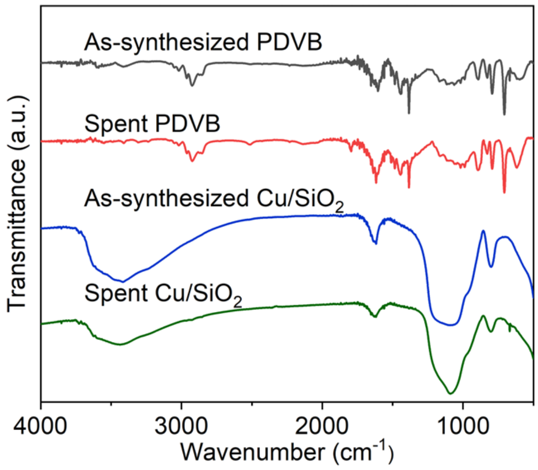

Figure S13. FTIR spectra of PDVB and Cu/SiO_2 components in the as-synthesized and the spent Cu/SiO_2-PDVB catalysts.

Note: As shown in Figure S13, the characteristic bands for PDVB[7] were not detected over the spent Cu/SiO_2 obtained from the Cu/SiO_2-PDVB catalyst, indicating the absence of the polymer fragments interacting with the Cu/SiO_2 surface, which supports the excellent stability of PDVB during catalysis.

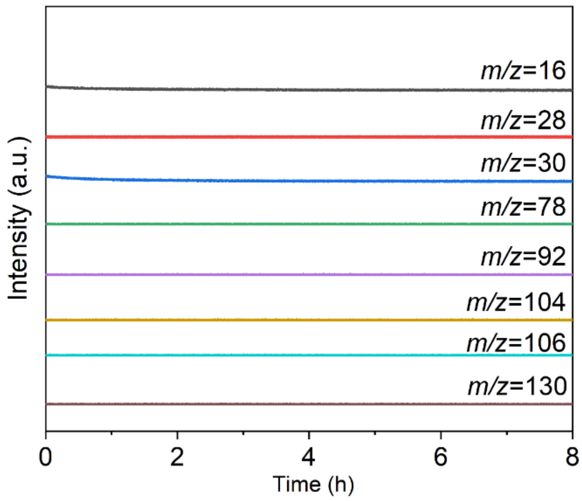

Figure S14. Mass spectra characterizing the effluent from the PDVB stability test at 240 °C for 8 h.

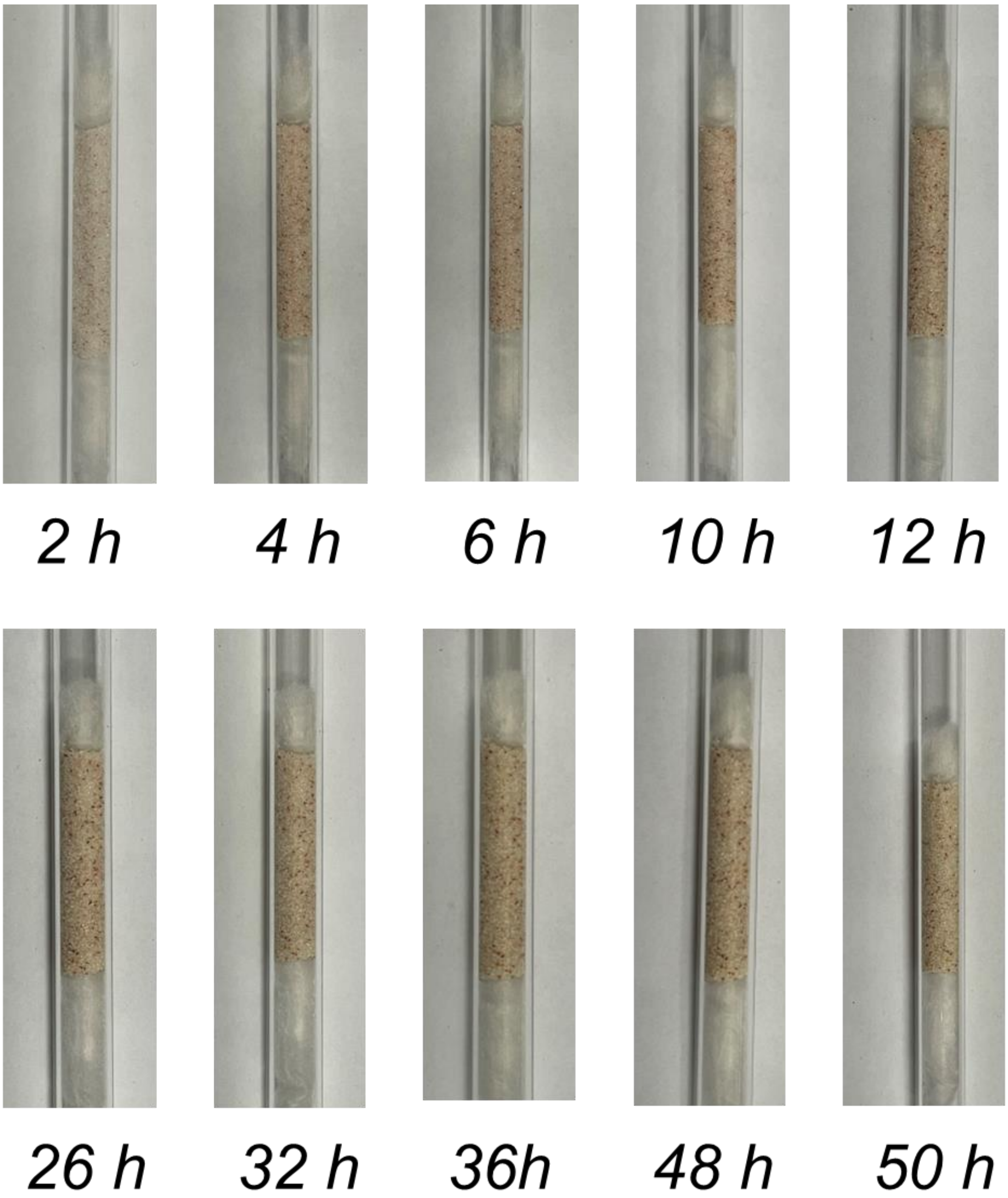

Figure S15. Photographs showing the PDVB granules in a quartz tube with thermal treatment at 240 °C for different periods. The photographs at 2, 26, and 50 h are also shown in the main text.

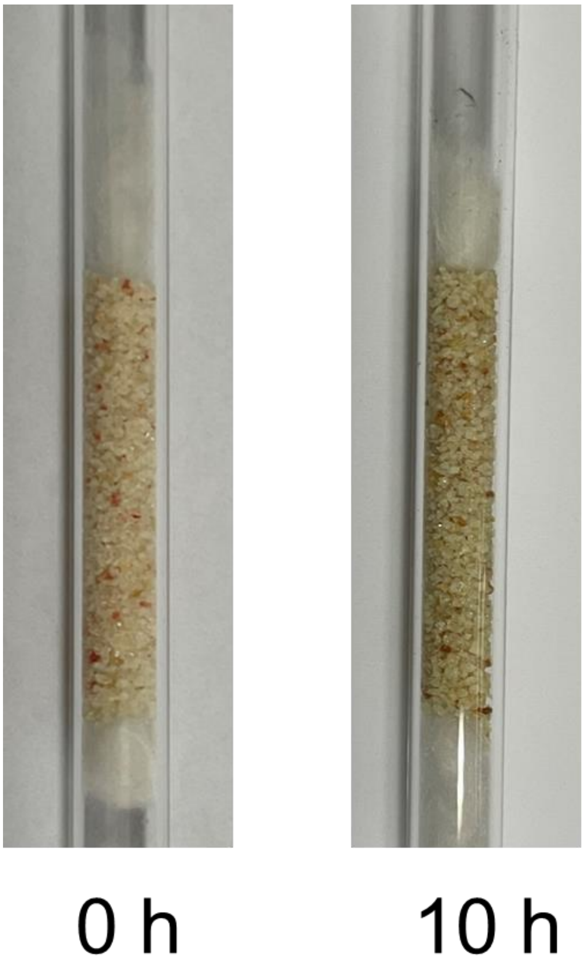

Figure S16. Photographs showing the PDVB granules in a quartz tube with thermal treatment at 300 °C for 10 h.

Note: This figure showed the photographs of the PDVB granules during heating treatment at 300 °C for 10 h, giving the well-maintained granule shape to exclude the possibility of its melting at 300 °C.

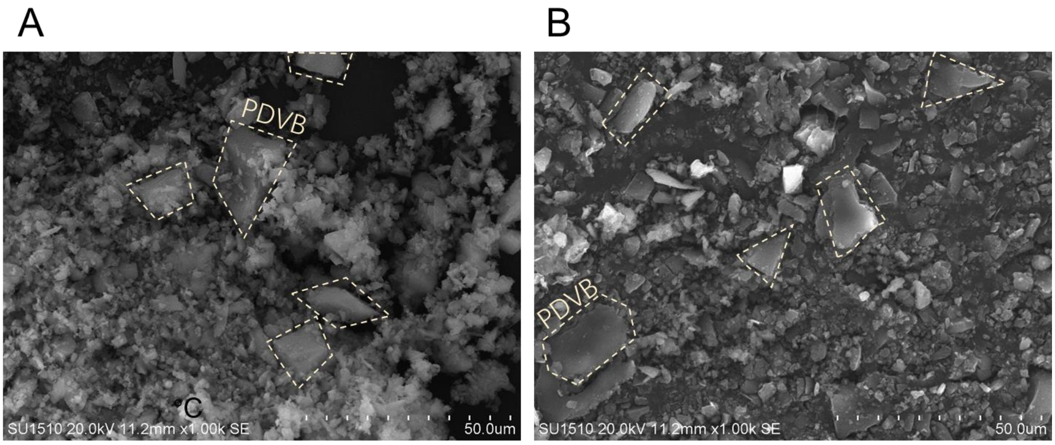

Figure S17. SEM images of (A) as-synthesized and (B) spent Cu/SiO_2-PDVB.

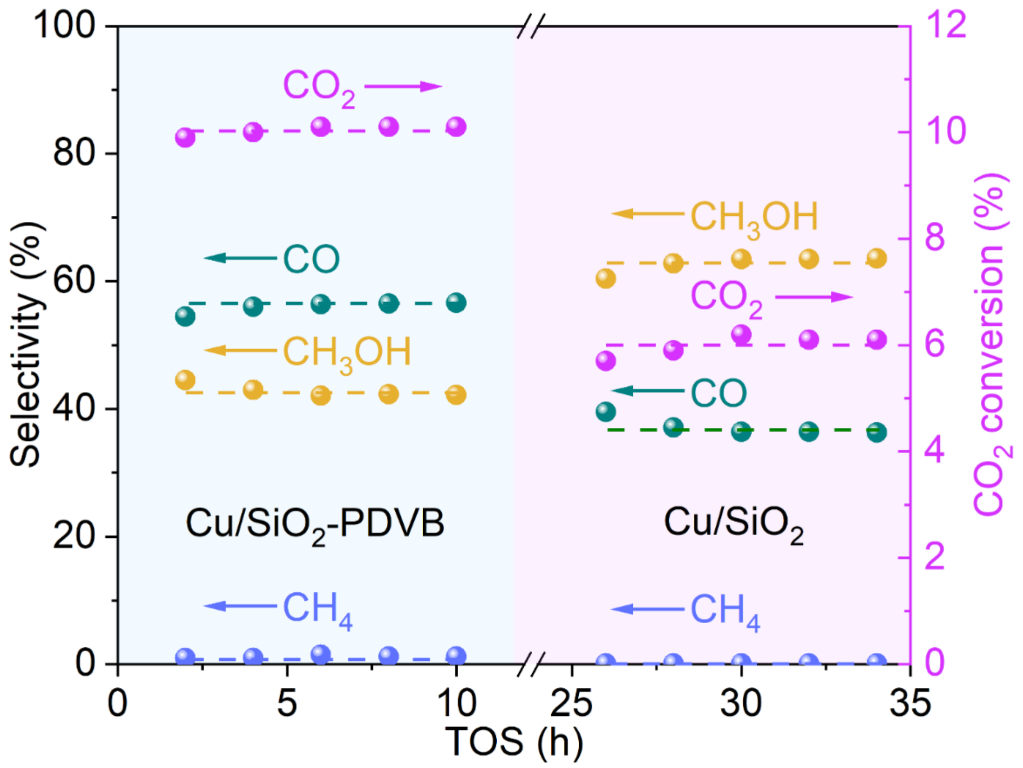

Figure S18. Catalytic performance of (left) Cu/SiO_2-PDVB and (right) Cu/SiO_2 after removing the PDVB component of spent Cu/SiO_2-PDVB in CO_2 hydrogenation. Reaction conditions: 3 MPa, 240 °C, SV of 6000 mL g_{cat}^{-1} h^{-1}, a feed gas of H_2/CO_2/Ar at 72/24/4 *vol%*.

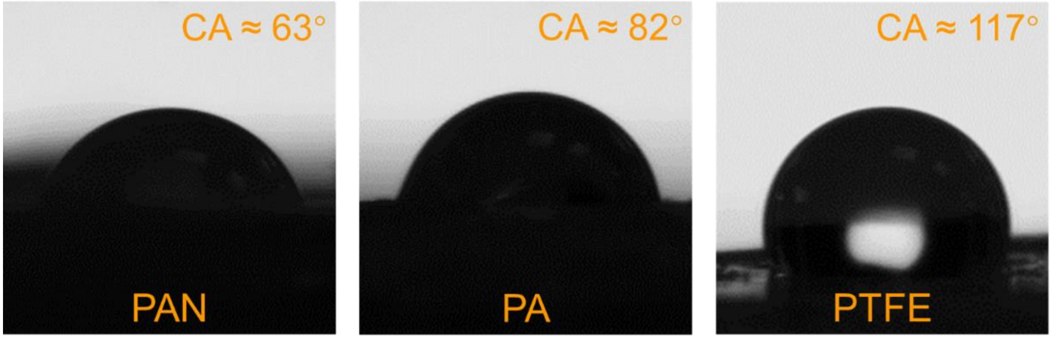

Figure S19. Water-droplet contact angles of PAN, PA, and PTFE.

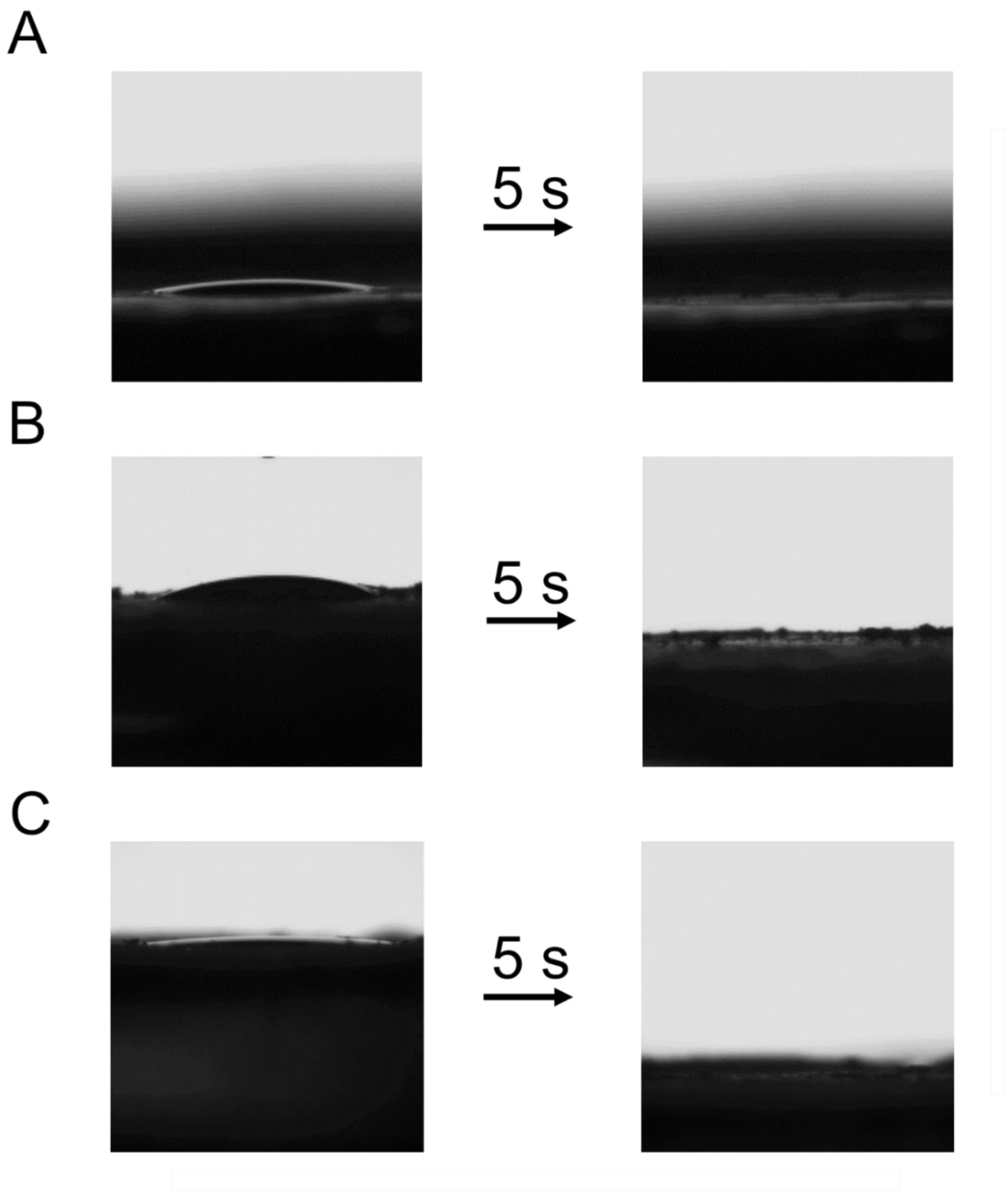

Figure S20. Water-droplet contact angles of (A) S-1 zeolite, (B) SiO_2, and (C) TiO_2 samples.

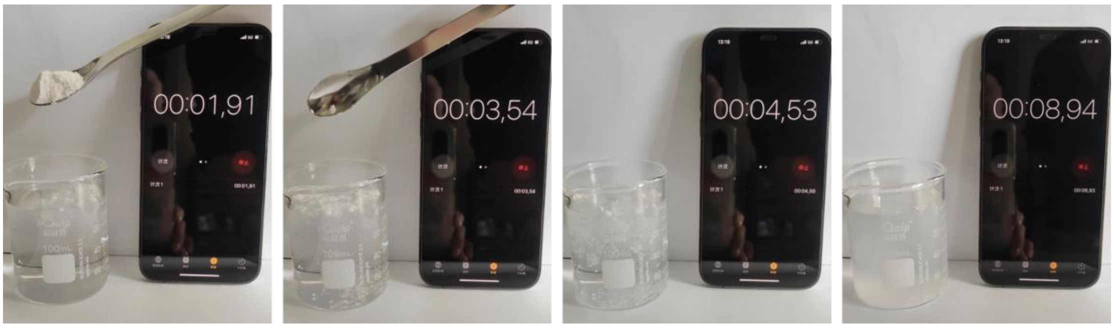

Figure S21. Photographs showing the process of S-1 zeolite dispersing in water. The cellphone showed the time for collecting the photographs at ~1.9, ~3.5, ~4.5, and ~8.9 seconds.

Note: Some siliceous zeolites are hydrophobic as reported previously,[24] but some others are hydrophilic, which are determined by the number of silanol groups/defects on the framework.[25] The silanol-rich siliceous zeolite has strong hydrophilicity.[26] In this work, the siliceous MFI zeolite was commercially obtained from Nankai Catalyst Co. in China, which has abundant silanol groups to make it hydrophilic. Figure S21 gave the photographs of S-1 zeolite in water. Clearly, it rapidly dispersed in water within 9 seconds rather than floating on the surface, which also supports its hydrophilicity.

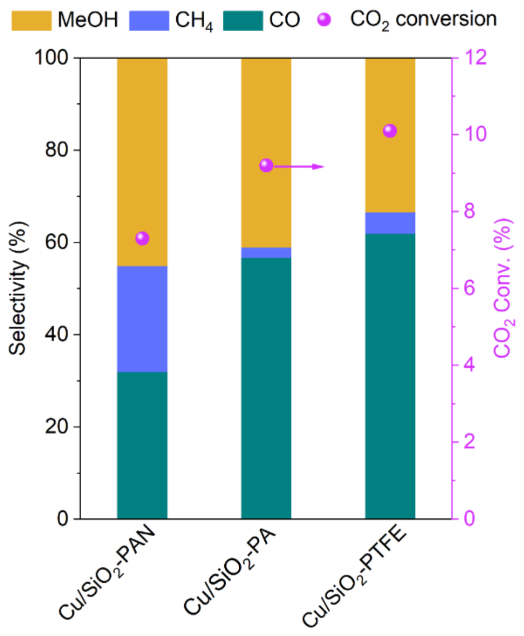

Figure S22. Data showing the performance of various catalysts in CO_2 hydrogenation. Reaction conditions: 3 MPa, 240 °C, SV of 6000 mL g_{cat}^{-1} h^{-1}, a feed gas of H_2/CO_2/Ar at 72/24/4 *vol%*.

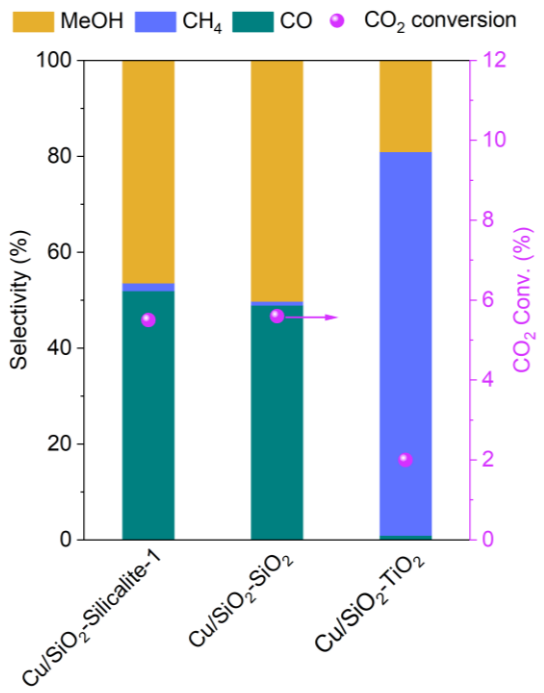

Figure S23. Data showing the performance of various catalysts in CO_2 hydrogenation. Reaction conditions: 3 MPa, 240 °C, SV of 6000 mL g_{cat}^{-1} h^{-1}, a feed gas of $H_2/CO_2/Ar$ at 72/24/4 *vol%*.

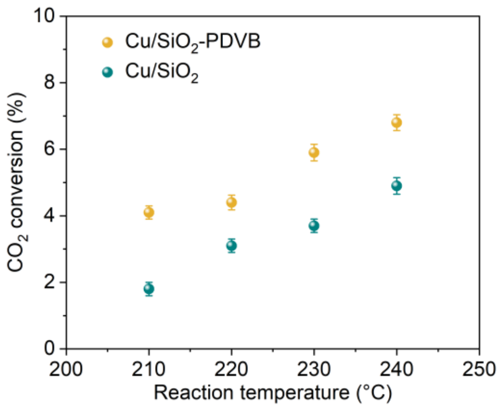

Figure S24. Data showing the catalytic performance of Cu/SiO_2 and Cu/SiO_2-PDVB in CO_2 hydrogenation at different temperatures. Reaction conditions: 3 MPa, 210-240 °C, SV of 18000 mL g_{cat}^{-1} h^{-1}, a feed gas of H_2/CO_2/Ar at 72/24/4 *vol%*.

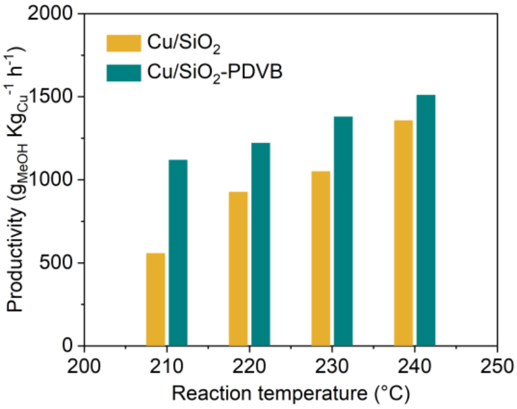

Figure S25. Data showing the methanol productivity of Cu/SiO_2 and Cu/SiO_2-PDVB catalysts in CO_2 hydrogenation. Reaction conditions: 3 MPa, 210-240 °C, SV of 18000 mL g_{cat}^{-1} h^{-1}, a feed gas of $H_2/CO_2/Ar$ at 72/24/4 *vol%*.

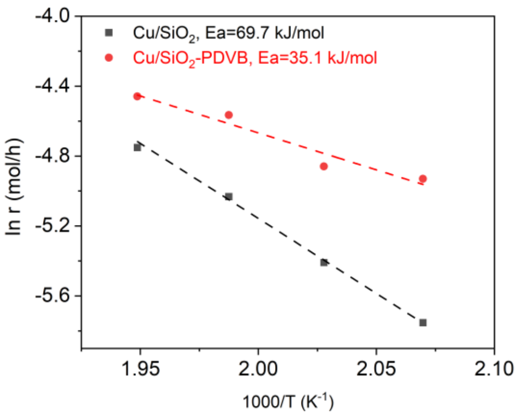

Figure S26. Experimental Arrhenius relationship (temperature dependencies of apparent activities) for CO_2 hydrogenation over Cu/SiO_2 and Cu/SiO_2-PDVB catalysts. Reaction conditions: 3 MPa, 210-240 °C, SV of 18000 mL g_{cat}^{-1} h^{-1}, a feed gas of H_2/CO_2/Ar at 72/24/4 *vol%*.

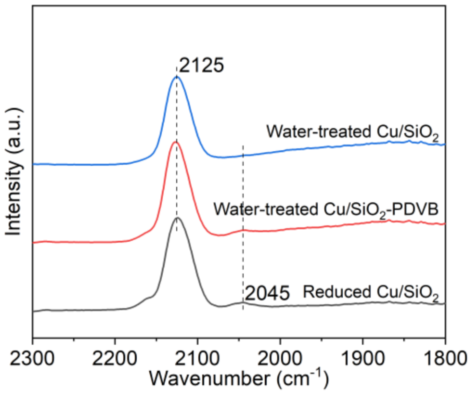

Figure S27. FT-IR spectra of CO adsorption over pre-reduced Cu/SiO_2 and pre-treated Cu/SiO_2 and Cu/SiO_2-PDVB.

Note: The as-reduced Cu/SiO_2 showed the CO adsorption signals at 2045 and 2125 cm^{-1}, which are assigned to the metallic Cu^0 and cationic $Cu^{\delta+}$ species, respectively.[27,28] After a water treatment, the Cu^0 signal disappeared on Cu/SiO_2 but was almost unchanged on Cu/SiO_2-PDVB.

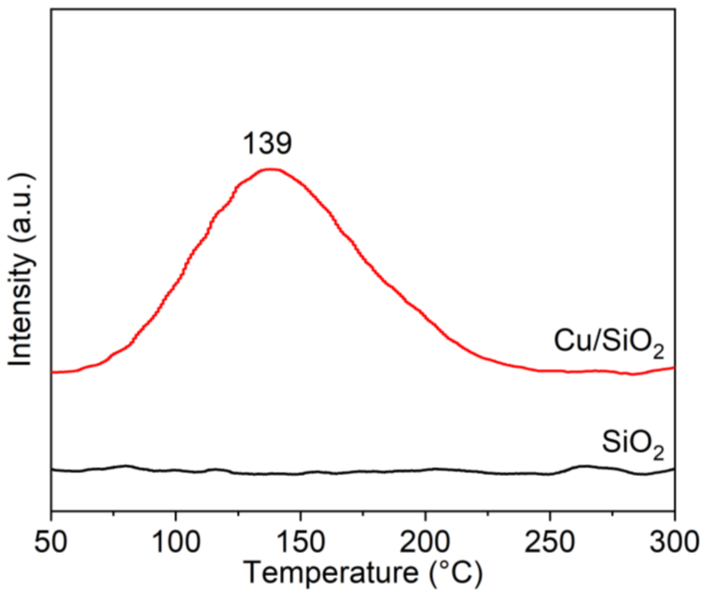

Figure S28. CO_2-TPD profiles of SiO_2 and Cu/SiO_2 samples.

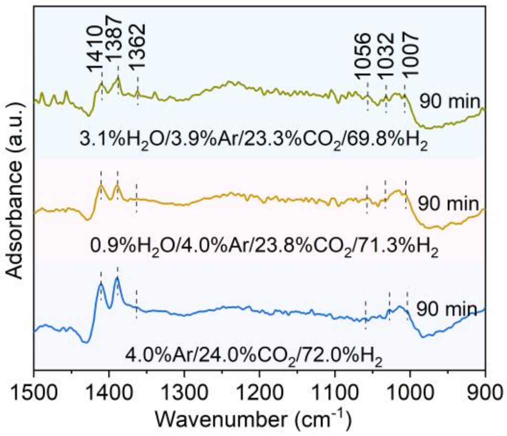

Figure S29. FT-IR spectra of Cu/SiO_2-PDVB in CO_2 hydrogenation with a feed gas of H_2/CO_2/Ar (72/24/4 *vol%*) at 240 °C.

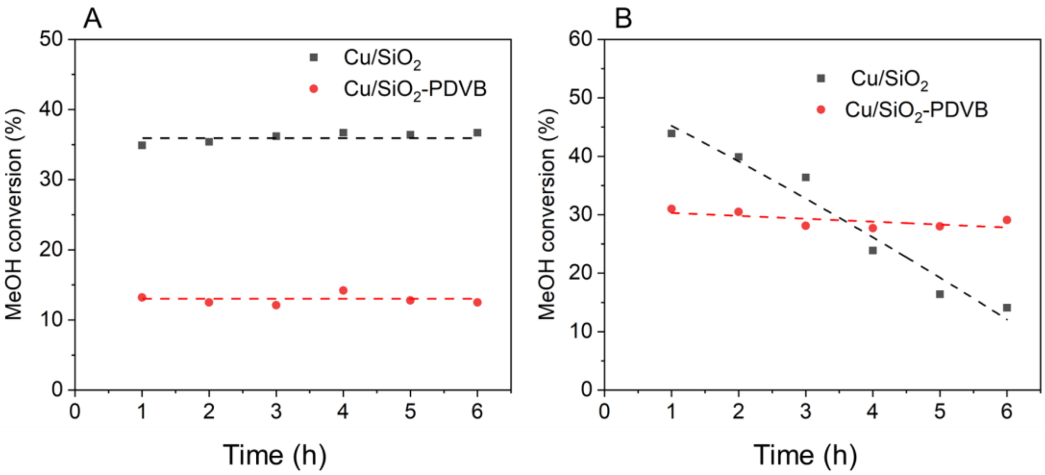

Figure S30. Data showing the catalytic performance of Cu/SiO_2 and Cu/SiO_2-PDVB catalysts in (A) methanol decomposition (7.4%CH_3OH/0.9%CH_4/91.7%N_2) and (B) methanol decomposition with feed containing water (4.2%CH_3OH/1.8%H_2O/0.9%CH_4/93.1%N_2). Reaction conditions: 240 °C, ambient pressure, SV of 12000 mL g_{cat}^{-1} h^{-1}, CH_3OH/CH_4/N_2 at 7.4/0.9/91.7 *vol%* and CH_3OH/H_2O/CH_4/N_2 at 4.2/1.8/0.9/93.1 *vol%*, the SV (mL g_{cat}^{-1} h^{-1}) was calculated according to the weight of Cu/SiO_2 catalyst amount in the reactor, the quartz sand and PDVB promoter were not considered. Cu/SiO_2-PDVB represents the catalyst in a powder mixing manner (the Cu/SiO_2 powder was mixed with an equivalent weight of PDVB powder, and then squeezed and crushed into granules with 20-40 mesh size for tests).

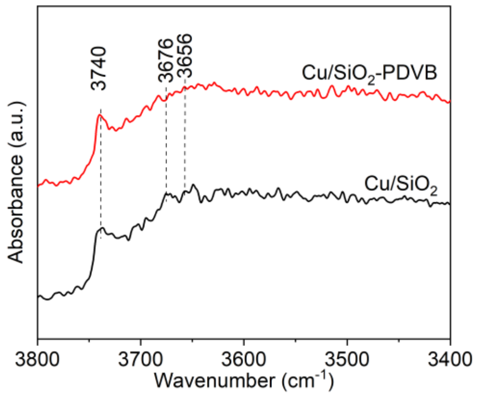

Figure S31. FT-IR spectra of spent Cu/SiO_2 and Cu/SiO_2-PDVB after methanol composition containing water.

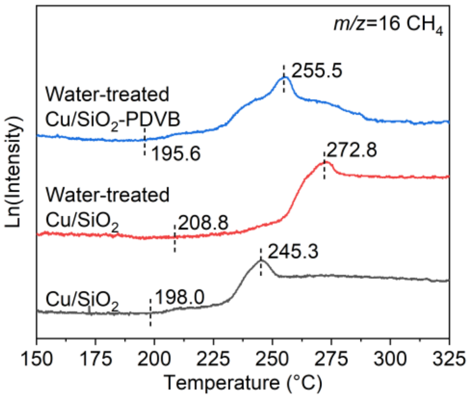

Figure S32. Data showing the methanol TPSR profiles of Cu/SiO_2, water-pretreated Cu/SiO_2, and water-pretreated Cu/SiO_2-PDVB catalysts. Conditions for the test: Cu/SiO_2 or Cu/SiO_2-PDVB catalyst were reduced by 10%H_2/Ar (20 mL min^{-1}) at 300 °C for 1 h and subsequently purged by 1 *vol%* water in Ar (20 mL min^{-1}) at 240 °C for 2 h.

Note: A very small amount of methane was formed on the Cu/SiO_2-PDVB catalyst while it was undetectable on the PDVB-free catalyst. This phenomenon is due to the different states of Cu species on these catalysts during the reaction. To confirm this hypothesis, we performed the temperature-programmed surface reaction of methanol on the Cu/SiO_2 catalyst. The as-reduced Cu/SiO_2 catalyst exhibited a CH_4 signal starting at 198.0 °C and reaching the maximum at 245.3 °C (Figure S32). After the water steaming treatment, the CH_4 signal shifted towards higher temperatures with an initial temperature of 208.8 °C and a maximum at 272.8 °C, indicating that the catalytic activity for converting methanol into CH_4 is hindered after water pretreatment, which is probably due to the oxidation of Cu^0 by water. Under the equivalent treatment on Cu/SiO_2-PDVB catalyst, the methane was still generated at 195.6 °C and reached a maximum at 255.5 °C, which is close to the results over the Cu/SiO_2 catalyst without water pretreatment, suggesting that PDVB could quickly ship the water from the Cu^0 surface and thus maintain Cu^0 statement against oxidation by water. This feature could enhance the performances in CO_2 hydrogenation to methanol with the formation of a little methane by the deep hydrogenation.

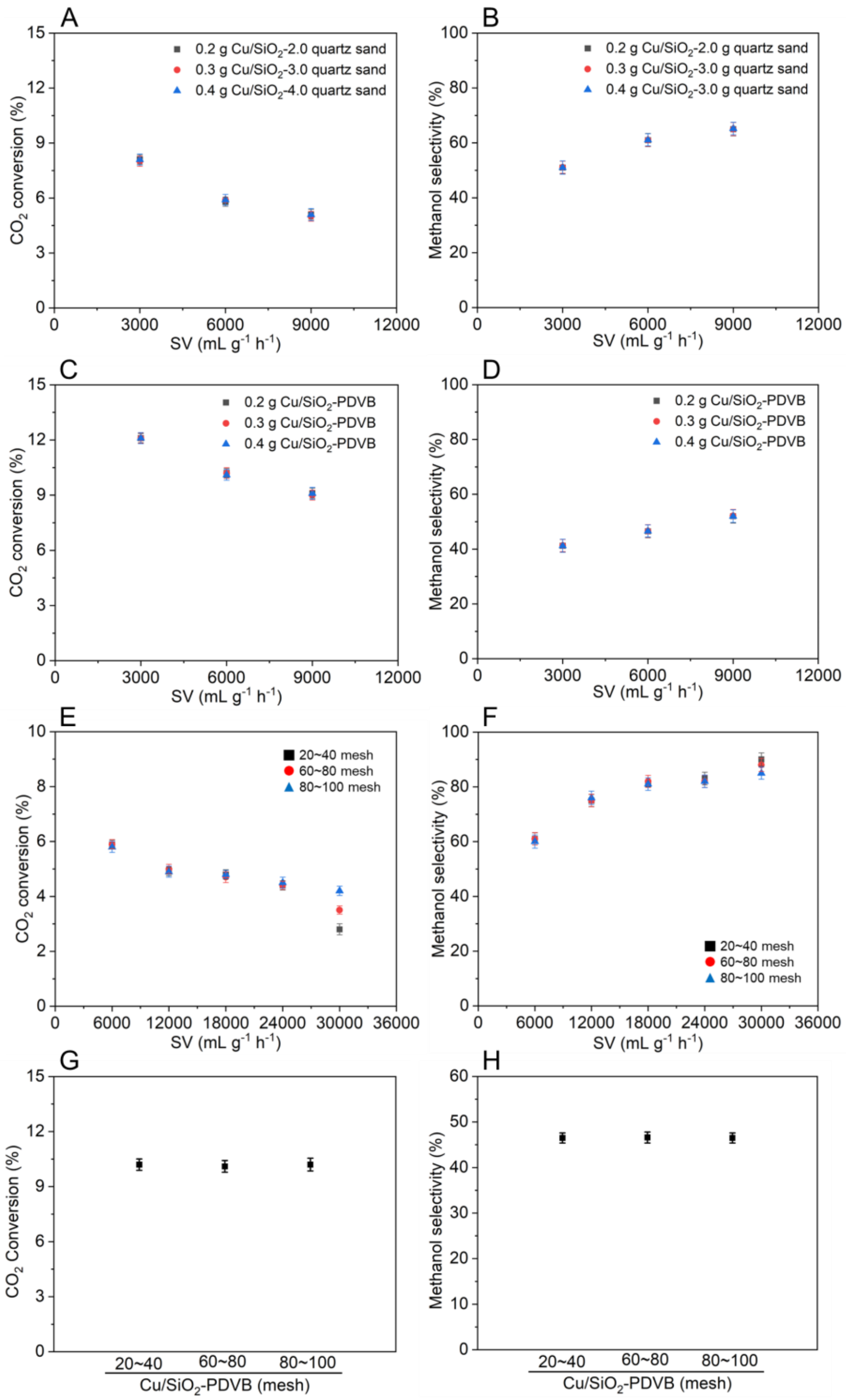

Figure S33. Data showing the (A) CO_2 conversion and (B) methanol selectivity of the Cu/SiO_2-quartz sand with different weight ratios of the Cu/SiO_2 to quartz sand and (C) CO_2 conversion and (D) methanol selectivity of the Cu/SiO_2-PDVB under varied SV in catalyzing CO_2 hydrogenation. (E) CO_2 conversion and (F) methanol selectivity of the Cu/SiO_2 with 20-40, 60-80, and 80-100 mesh under varied SV in catalyzing CO_2 hydrogenation. (G) CO_2 conversion and (H) methanol selectivity of the Cu/SiO_2-PDVB under varied mesh size in CO_2 hydrogenation. Reaction conditions: 3 MPa, 240 °C, SV of 6000-30000 mL g_{cat}^{-1} h^{-1}, a feed gas of H_2/CO_2/Ar ratio at 72/24/4 *vol%*. The SV (mL g_{cat}^{-1} h^{-1}) was calculated according to the weight of the Cu/SiO_2 catalyst amount in the reactor, the quartz sand and PDVB were not considered.

Note: In Figures S33A and 33B, symbol ■ represents the catalytic data over a mixture of Cu/SiO_2 granules (0.2 g, 20-40 mesh) and quartz sand granules (2.0 g, 20-40 mesh), and when the flow rates of feed gas are 10, 20 and 30 mL min^{-1}, the corresponding SV reach 3000, 6000, and 9000 mL g_{cat}^{-1} h^{-1}, respectively.

Symbol ● represents the catalytic data over a mixture of Cu/SiO_2 granules (0.3 g, 20-40 mesh) and quartz sand granules (3.0 g, 20-40 mesh), and when the flow rates of feed gas are 15, 30, and 45 mL min^{-1}, the corresponding SV reaches 3000, 6000, and 9000 mL g_{cat}^{-1} h^{-1}, respectively.

Symbol ▲ represents the catalytic data over a mixture of Cu/SiO_2 granules (0.4 g, 20-40 mesh) and quartz sand granules (4.0 g, 20-40 mesh), and when the flow rates of feed gas are 20, 40, and 60 mL min^{-1}, the corresponding SV reaches 3000, 6000, and 9000 mL g_{cat}^{-1} h^{-1}, respectively.

In Figures S33C and 33D, Cu/SiO_2-PDVB represents the catalyst with powder mixing manner (the Cu/SiO_2 powder was mixed with an equivalent weight of PDVB powder, and then squeezed and crushed into granules with 20-40 mesh size for tests).

Symbol ■ represents the Cu/SiO_2-PDVB catalyst with 0.2 Cu/SiO_2 and 0.2 g PDVB, the flow rates of feed gas are 10, 20, and 30 mL min^{-1}, the corresponding SV are 3000, 6000, and 9000 mL g_{cat}^{-1} h^{-1}, respectively.

Symbol ● represents the Cu/SiO_2-PDVB catalyst with 0.3 Cu/SiO_2 and 0.3 g PDVB, the flow rates of feed gas are 15, 30, and 45 mL min^{-1}, the corresponding SV are 3000, 6000, and 9000 mL g_{cat}^{-1} h^{-1}, respectively.

Symbol ▲ represents the Cu/SiO_2-PDVB catalyst with 0.4 Cu/SiO_2 and 0.4 g PDVB, the flow rates of feed gas are 20, 40, and 60 mL min^{-1}, the corresponding SV are 3000, 6000, and 9000 mL g_{cat}^{-1} h^{-1}, respectively.

In Figures S33E and 33F, symbol ■ represents the data over 0.2g of Cu/SiO_2 granules are in 20-40 mesh size, the flow rates of feed gas are 20, 40, 60, 80, and 100 mL min^{-1}, the SV are 6000, 12000, 18000, 24000, and 30000 mL g_{cat}^{-1} h^{-1}, respectively.

Symbol ● represents the data over 0.2g of Cu/SiO_2 granules are 60-80 mesh size, the flow rates of feed gas are 20, 40, 60, 80, and 100 mL min^{-1}, the SV are 6000, 12000, 18000, 24000, and 30000 mL g_{cat}^{-1} h^{-1}, respectively.

Symbol ▲ represents the data over 0.2g of Cu/SiO_2 granules are in 80-100 mesh size, the flow rates of feed gas are 20, 40, 60, 80, and 100 mL min^{-1}, the SV are 6000, 12000, 18000, 24000, and 30000 mL g_{cat}^{-1} h^{-1}, respectively.

In Figures S33G and 33H, Cu/SiO_2-PDVB represents the catalyst with powder mixing manner (the Cu/SiO_2 powder was mixed with an equivalent weight of PDVB powder, and then squeezed and crushed into granules with 20-40, 60-80, and 80-100 mesh size for tests).

In some cases, the apparent E_a could be influenced by the mass transfer limitation. To exclude these issues, we have performed several CO_2 hydrogenation reactions to investigate the effect of mass transfer limitations on catalytic performance. External mass transfer limitation always causes the fluctuation of conversion rate due to the change in gas mass flow rate. Therefore, we designed several CO_2 hydrogenation reactions, which were carried out at the same SV of 3000, 6000, and 9000 mL $g_{cat}^{-1}h^{-1}$ with varied flow rates and catalysts, e.g., 20, 30, 40 mL min^{-1} feed gas for 0.2 g of Cu/SiO_2 catalyst diluted with 2.0 g quartz sands, 0.3 g Cu/SiO_2 of Cu/SiO_2 catalyst diluted with 3.0 g quartz sands, and 0.4 g of Cu/SiO_2 catalyst diluted with 4.0 g of quartz sands. As shown in Figures S33A and 33B, all three catalysts gave similar CO_2 conversion and methanol selectivity at 8.1%, 5.9%, 5.1%, and 51.0%, 61.3%, 65.0% under the SV of 3000, 6000, and 9000 mL g_{cat}^{-1} h^{-1}, respectively. Because these conversion rate does not change with the fluctuation of the mass flow rate, the external mass transfer limitation can be neglected over Cu/SiO_2 catalyst. Under the equivalent tests, similar results were obtained in the tests over

the Cu/SiO_2-PDVB catalyst (Figures S33C and 33D), which also excluded the external mass transfer limitation in the catalyst containing PDVB.

Internal mass transfer limitation is closely related to the catalyst granule sizes and flow rate. Therefore, we designed several CO_2 hydrogenation reactions, which were carried out under the same SV of 6000, 12000, 18000, 24000, and 30000 mL g_{cat}^{-1} h^{-1} with varied catalyst granule sizes, e.g., the CO_2 hydrogenation reaction was carried out under SV at 6000 mL g_{cat}^{-1} h^{-1} with catalyst size of 20-40, 60-80, 80-100 mesh, respectively. As seen in Figure S12, the CO_2 conversion and methanol selectivity were constant at ~5.9% and ~61.3%, which could avoid the internal mass transfer limitation with granule sizes ranging from 20-100 mesh under the given reaction conditions. Furthermore, we increased the flow rate from 12000 to 24000 mL g_{cat}^{-1} h^{-1}, and the CO_2 conversion and methanol selectivity keep constant at under 4.9%, 4.8%, 4.5%, and 75.0%, 81.2%, 83.0%, respectively, over Cu/SiO_2 with these three different granule sizes. Only when the SV reaches as high as 30000 mL g_{cat}^{-1} h^{-1}, the Cu/SiO_2 catalysts with granule sizes of 20-40, 60-80 and 80-100 mesh gave varied CO_2 conversion of 2.8%, 3.5, and 4.2%, respectively (Figures S33E and 33F). That is, in our study under such mild reaction conditions with SV at 6000 mL g_{cat}^{-1} h^{-1}, much lower than 30000 mL g_{cat}^{-1} h^{-1}, the conversion rate does not change with the changing of the granule sizes in the range of 20-100 mesh. A similar result was also obtained in the tests over the Cu/SiO_2-PDVB catalyst (Figures S33G and 33H). Therefore, we can rule out the effect of internal mass transfer limitation on the catalytic performance over Cu/SiO_2 and Cu/SiO_2-PDVB catalysts. The different E_a should be due to the different active sites after mixing Cu/SiO_2 with PDVB more than the diffusion limitation factors.

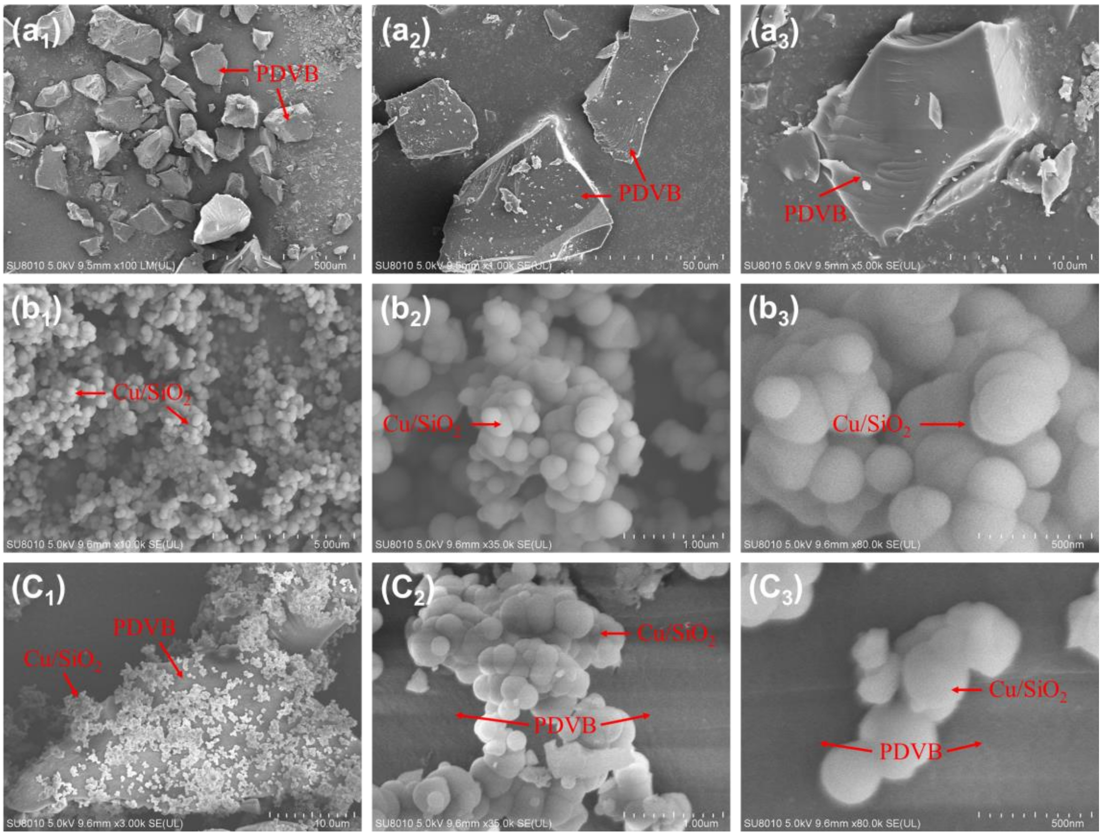

Figure S34. SEM images of (a) PDVB, (b) Cu/SiO_2, and (c) Cu/SiO_2-PDVB mixture.

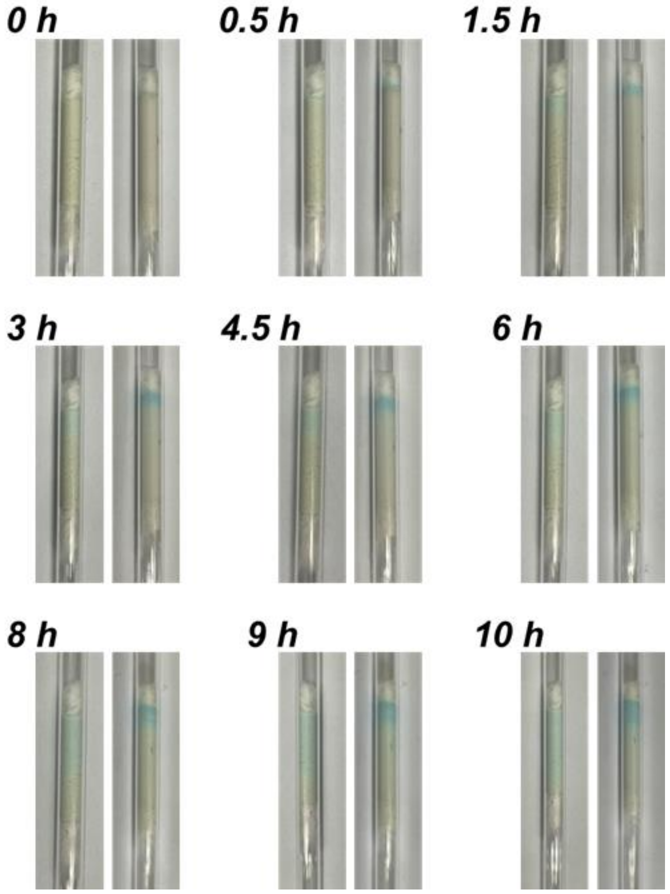

Figure S35. Photographs of $CuSO_4$ mixed with PDVB (left) and $CuSO_4$ mixed with quartz powder (right) in H_2O/N_2 flow (25 mL min^{-1}) at room temperature for different periods.

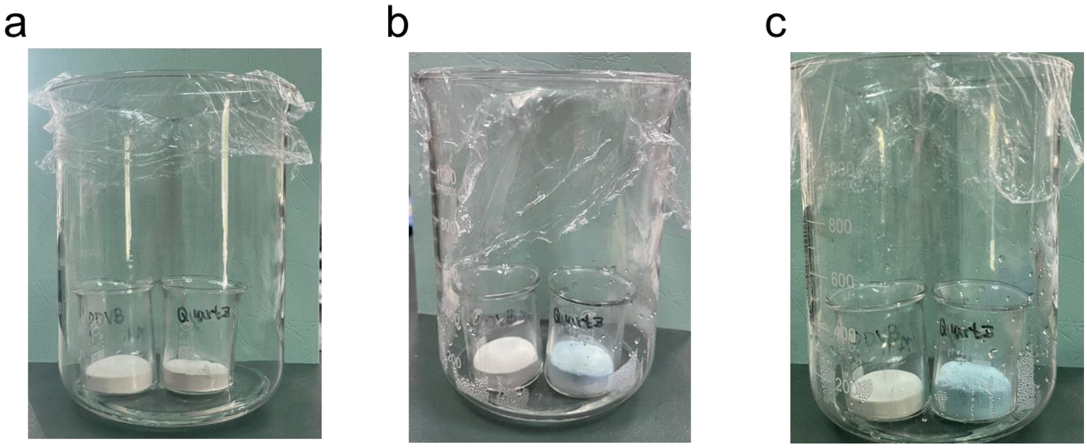

Figure S36. Photographs showing the $CuSO_4$ mixed with PDVB (left) and $CuSO_4$ mixed with quartz powder (right) in a confined beaker containing water for different periods of (a) 0, (b) 12, and (c) 36 hours. The test was mostly performed at room temperature, but the beaker was heated to 60 °C for 2 hours in each 12 hours during the test.

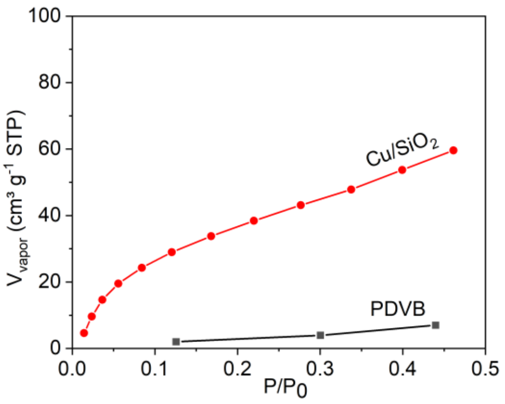

Figure S37. Water adsorption test over PDVB and Cu/SiO_2.

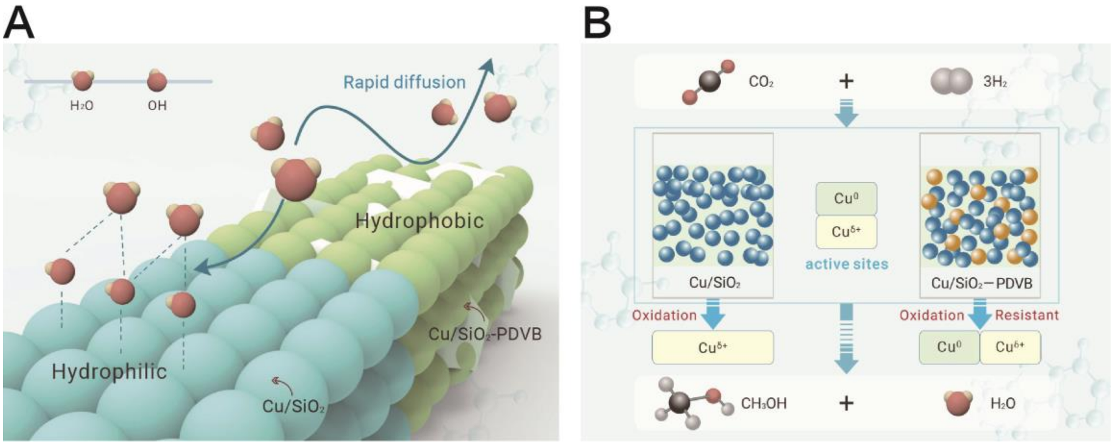

Figure S38. Scheme showing (A) the effect of PDVB on water diffusion on the catalyst surface and (B) the oxidation of copper species over Cu/SiO_2 and hindered copper oxidation over Cu/SiO_2-PDVB with water during CO_2 hydrogenation reaction.

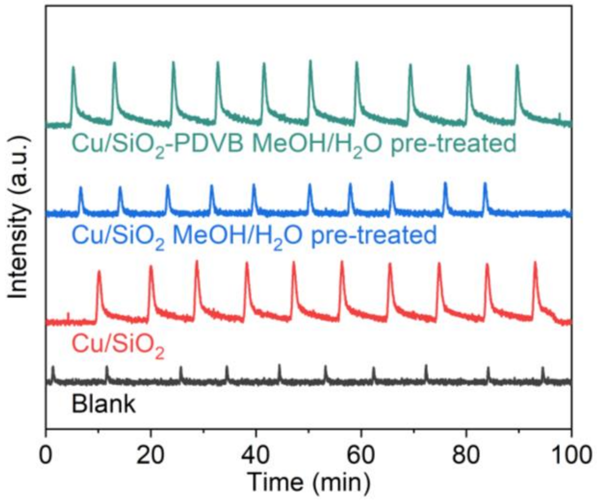

Figure S39. H-D exchange test characterizing the hydrogenation activity of various catalysts.

Note: We performed a pulse experiment to explore the activity of various catalysts in the H-D exchange, which was usually related to the hydrogenation activity in many reactions. The catalysts were localized within the flowing H_2 (10% in Ar) atmosphere at 240 °C, and the D_2 (10% in Ar) was pulsed periodically. The HD (m/z at 3) signals in the emission gas were detected by a mass spectrometer (MS). The Cu/SiO_2 exhibited strong HD signals in the test, suggesting high activity for H-D exchange. After a methanol/water treatment, the HD signals were obviously weakened, suggesting partial deactivation. The metallic Cu species were usually regarded as active sites for hydrogenation.[29-31] Therefore, the deactivation could be explained by the oxidation of the Cu surface by the water/methanol treatment. Under the equivalent treatment, the HD signals over the Cu/SiO_2-PDVB catalyst were still very strong, confirming the oxidation resistance that is in good agreement with the results of the FTIR study.

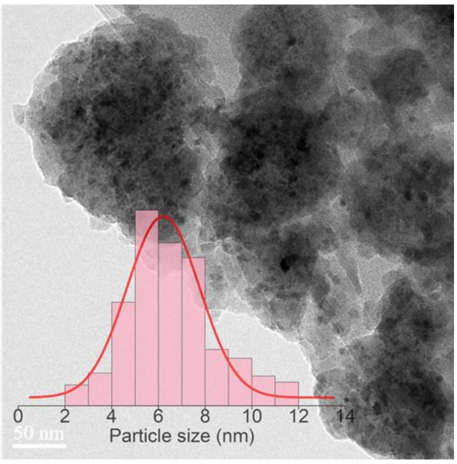

Figure S40. TEM images and the corresponding Cu nanoparticle size distribution of spent Cu/SiO_2 catalyst after the test in CO_2 hydrogenation.

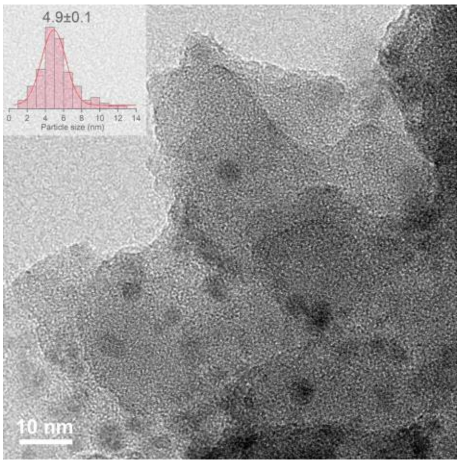

Figure S41. TEM images and the corresponding Cu nanoparticle size distribution of spent Cu/SiO_2-PDVB catalyst after the test in CO_2 hydrogenation.

Note: Compared with the spent Cu/SiO_2, the spent Cu/SiO_2-PDVB exhibited slightly smaller Cu nanoparticles (4.9 vs 6.0 nm), which might be because that the PDVB weakened the water-triggered Cu sintering during the catalysis. It has been previously identified that slightly increasing the copper nanoparticle size over the catalysts (e.g. <8.0 nm) could not obviously affect the apparent catalytic performance in some cases,[32-34] which might explain the constant durability of the catalyst in CO_2 hydrogenation.

References

[1] B. An, J. Zhang, K. Cheng, P. Ji, C. Wang, W. Lin, *J. Am. Chem. Soc.* **2017**, *139*, 3834−3840.
[2] W. Zhou, K. Cheng, J. Kang, C. Zhou, V. Subramanian, Q. Zhang, Y. Wang, *Chem. Soc. Rev.* **2019**, *48*, 3193-3228.
[3] Z. Wang, Z. Xu, S. Peng, M. Zhang, G. Lu, Q. Chen, Y. Chen, G. Guo, *ACS Catal.* **2015**, *5*, 4255–4259.
[4] Y. Zhang, L. Zhong, H. Wang, P. Gao, X. Li, S. Xiao, G. Ding, W. Wei, Y. Sun, *J. CO_2 Util.* **2016**, *15*, 72−82.
[5] H. Zhan, F. Li, P. Gao, N. Zhao, F. Xiao, W. Wei, L. Zhong, Y. Sun, *J. Power Sources* **2014**, *251*, 113-121.
[6] L. Li, D. Mao, J. Yu, X. Guo, *J. Power Sources* **2015**, *279*, 394-404.
[7] X. Fang, Y. Men, F. Wu, Q. Zhao, R. Singh, P. Xiao, T. Du, P. A. Webley, *Chem. Eng. J.* **2019,** *378*, 122052.
[8] X. Hu, W. Qin, Q. Guan, W. Li, *ChemCatChem* **2018**, *10*, 4438–4449.
[9] G. Noh, E. Lam, J. L. Alfke, K. Larmier, K. Searles, P. Wolf, C. Coperet, *ChemSusChem* **2019**, *12*, 968−972.
[10] T. Witoon, S. Bumrungsalee, M. Chareonpanich, J. Limtrakul, *Energy Convers. Manage.* **2015**, *103*, 886−894.
[11] H. Yang, P. Gao, C. Zhang, L. Zhong, X. Li, S. Wang, H. Wang, W. Wei, Y. Sun, *Catal. Commun.* **2016**, *84*, 56−60.
[12] J. Yu, M. Yang, J. Zhang, Q. Ge, A. Zimina, T. Pruessmann, L. Zheng, J.-D. Grunwaldt, J. Sun, *ACS Catal.* **2020**, *10*, 14694−14706.
[13] X. Jiang, N. Koizumi, X. Guo, C. Song, *Appl. Catal. B-Environ.* **2015**, *170-171*, 173-185
[14] Q. Jiang, Y. Liu, T. Dintzer, J. Luo, K. Parkhomenko, A.-C. Roger. *Applied Catal. B-Environ.* **2020**, *269*, 118804.
[15] J. Zuo, K. Chen, J. Zheng, L. Ye, Y. Yuan, *J. CO_2 Util.* **2021**, *52*, 101699.
[16] A. Jangam, P. Hongmanorom, M. H. Wai, A. J. Poerjoto, S. Xi, A. Borgna, S. Kawi, *ACS Appl. Energy Mater.* **2021**, *4*, 12149−12162.
[17] R.-P. Ye, Y. Chen, T. R. Reina, Z. Cao, T. Xu, X. Chen, Y. Jin, X. L. Zhang, J. Liu, *Adv. Energy Sustain. Res.* **2021**, *2*, 2100082.
[18] F. Arena, G. Mezzatesta, G. Zafarana, G. Trunfio, F. Frusteri, L. Spadaro, *J. Catal.* **2013**, *300*, 141–151
[19] G. Bonura, M. Cordaro, C. Cannilla, F. Arena, F. Frusteri, *Appl. Catal. B-Environ.* **2014**, *152*, 152–161.
[20] J. Zhang, L. Wang, B. Zhang, H. Zhao, U. Kolb, Y. Zhu, L. Liu, Y. Han, G. Wang, C. Wang, D. S. Su, B. C. Gates, F.-S. Xiao, *Nat. Catal.* **2018**, *1*, 540–546.
[21] L. Liu, M. Lopez-Haro, C. W. Lopes, S. Rojas-Buzo, P. Concepcion, R. Manzorro, L. Simonelli, A. Sattler, P. Serna, J. J. Calvino, A. Corma, *Nat. Catal.* **2020**, *3*, 628−638.
[22] W. Fang, C. Wang, Z. Liu, L. Wang, L. Liu, H. Li, S. Xu, A. Zheng, X. Qin, L. Liu, F.-S. Xiao, *Science* **2022**, *377*, 406–410.
[23] P. Gao, S. Li, X. Bu, S. Dang, Z. Liu, H. Wang, L. Zhong, M. Qiu, C. Yang, J. Cai, W. Wei, Y. Sun, *Nat. Chem.* **2017**, *9*, 1019–1024.
[24] E. M. Flanigen, J. M. Bennett, R. W. Grose, J. P. Cohen, R. L. Patton, R. M. Kirchner, J. V. Smith, *Nature* **1978**, *271*, 512-516.
[25] C. Wang, Z. Liu, L. Wang, X. Dong, J. Zhang, G. Wang, S. Han, X. Meng, A. Zheng, F.-S. Xiao, *ACS Catal.* **2018**, *8*, 474-481.
[26] D. T. Bregante, M. C. Chan, J. Z. Tan, E. Z. Ayla, C. P. Nicholas, D. Shukla, D. W. Flaherty, *Nat. Catal.* **2021**, *4*, 797-808
[27] D. Li, F. Xu, X. Tang, S. Dai, T. Pu, X. Liu, P. Tian, F. Xuan, Z. Xu, I. E. Wachs, M. Zhu, *Nat. Catal.* **2022**, *5*, 99–108.

[28] T. Venkov, K. Fajerwerg, L. Delannoy, H. Klimev, K. Hadjiivanov, C. Louis, *Appl. Catal. A-Gen.* **2006**, *301*, 106–14.
[29] A. Karelovic, G. Galdames, J. C. Medina, C. Yévenes, Y. Barra, R. Jiménez, *J. Catal.* **2019**, *369*, 415–426.
[30] Y. Yang, J. Evans, J. A. Rodriguez, M. G. White, P. Liu, *Phys. Chem. Chem. Phys.* **2010**, *12*, 9909–9917.
[31] R. Ladera, F. J. Pérez-Alonso, J. M. González-Carballo, M. Ojeda, S. Rojas, J. L. G. Fierro, *Appl. Catal. B-Environ.* **2013**, *142*, 241–248.
[32] R. Beerthuis, J. W. de Rijk, J. M. S.Deeley, G. J. Sunley, K. P. de Jong, P, E. de Jongh, *J. Catal.* **2020**, *388*, 30–37.
[33] H. Lyu, B. Hu, G. Liu, X. Hong, L. Zhuang, *Acta Phys.-Chim. Sin.* **2020**, *36*, 1911008.
[34] M. Samim, N. K. Kaushik, A. Maitra, *Bull. Mater. Sci.* **2007**, *30*, 535–540.
